# YY1 binding association with sex-biased transcription revealed through X-linked transcript levels and allelic binding analyses

**DOI:** 10.1038/srep37324

**Published:** 2016-11-18

**Authors:** Chih-yu Chen, Wenqiang Shi, Bradley P. Balaton, Allison M. Matthews, Yifeng Li, David J. Arenillas, Anthony Mathelier, Masayoshi Itoh, Hideya Kawaji, Timo Lassmann, Yoshihide Hayashizaki, Piero Carninci, Alistair R. R. Forrest, Carolyn J. Brown, Wyeth W. Wasserman

**Affiliations:** 1 Centre for Molecular Medicine and Therapeutics, Child and Family Research Institute, University of British Columbia, Vancouver, British Columbia, Canada; 2 Graduate Program in Bioinformatics, University of British Columbia, Vancouver, British Columbia, Canada; 3 Department of Medical Genetics, University of British Columbia, Vancouver, British Columbia, Canada; 4 RIKEN Omics Science Center, Yokohama, Japan; 5 RIKEN Center for Life Science Technologies, Division of Genomic Technologies, Yokohama, Japan; 6 RIKEN Preventive Medicine and Diagnosis Innovation Program, Wako, Saitama, Japan; 7 Harry Perkins Institute of Medical Research, QEII Medical Centre and Centre for Medical Research, the University of Western Australia, Nedlands, Western Australia, Australia

## Abstract

Sex differences in susceptibility and progression have been reported in numerous diseases. Female cells have two copies of the X chromosome with X-chromosome inactivation imparting mono-allelic gene silencing for dosage compensation. However, a subset of genes, named escapees, escape silencing and are transcribed bi-allelically resulting in sexual dimorphism. Here we conducted *in silico* analyses of the sexes using human datasets to gain perspectives into such regulation. We identified transcription start sites of escapees (escTSSs) based on higher transcription levels in female cells using FANTOM5 CAGE data. Significant over-representations of YY1 transcription factor binding motif and ChIP-seq peaks around escTSSs highlighted its positive association with escapees. Furthermore, YY1 occupancy is significantly biased towards the inactive X (Xi) at long non-coding RNA loci that are frequent contacts of Xi-specific superloops. Our study suggests a role for YY1 in transcriptional activity on Xi in general through sequence-specific binding, and its involvement at superloop anchors.

Sex disparities in disease progression and susceptibility for many diseases, including cancer^1^, autism^2^, cardiac^3^ and autoimmune disorders^4^, have long been known. Such discrepancies likely result from a combination of the sex chromosomes, sex hormones, and environmental factors. Due to such sexual dimorphism, there has been a recent push in policy to balance sex in cell and animal studies by NIH^5^. The key genetic differences between the sexes are the sex chromosomes, with mammalian females being XX and males XY. Furthermore, there is an enrichment of brain-related genes on the X chromosome in mammals^6^. With the exception of the pseudoautosomal regions (PAR) shared with the Y-chromosome, X-linked genes are present in two copies in females and only one in males. X-chromosome inactivation (XCI) silences one copy of the X chromosome (chrX) in female cells in order to compensate for dosage between the sexes. Up-regulation of *XIST*, a long non-coding RNA (lncRNA), is known to be responsible for initiation of XCI, and this process has been reported to be mediated through recruitment of factors such as polycomb repressive complex 2, leading to tri-methylation of histone H3 at lysine 27 (H3K27me3) and silencing of the inactive X chromosome (Xi)^7^. XCI involves the establishment of a peripheral nuclear architecture, which includes association with the lncRNA, *FIRRE*^8^, anchoring the Xi near the nucleolus to preserve H3K27me3 and the silencing state^9^. DNA methylation (DNAm) is recruited to promoters, providing maintenance of the inactive state. As a result, the majority of chrX genes outside of PAR regions are subject to XCI, and are transcribed mono-allelically from the active X (Xa) in female somatic cells. A small subset of chrX genes including *XIST,* known as escapees, escape from XCI and are transcribed on the Xi^10^. These escapees are therefore bi-allelically transcribed with the exception of *XIST*, which is solely transcribed from the Xi. Binding of the Ying-Yang 1 (YY1) transcription factor (TF) to *XIST* RNA and DNA contributes to *XIST* transcription^11^,^12^,^13^. *XIST* and *FIRRE* are among the four long non-coding RNAs (lncRNAs) previously found at frequently interacting regions of Xi-specific superloops in the GM12878 cell line^14^. Rao *et al*. reported tandem CTCF motifs at three (*FIRRE, DXZ4, LOC550643*) of the four lncRNAs, and suggested a role for chromatin looping through CTCF and RAD21 in shaping the chromatin structure of Xi. At the *FIRRE* locus, Yang *et al*. showed differential occupancy by CTCF and YY1 using ENCODE ChIP-seq peaks from male and female cells^9^.

Investigation of differences between the sexes has been tackled both through direct comparisons of male and female data and/or a focused study of XCI. Direct sex comparisons have identified male:female differences in gene expression, DNAm, accessible chromatin, and TF binding levels. At the gene expression level, microarray and RNA-seq platforms have been used to examine differential gene expression between the sexes. The Genotype-Tissue Expression (GTEx) pilot study reported genes with differential expression between male and female samples in 43 tissues using RNA-seq data^15^. Sex differences in brain expression have been observed repeatedly^16^,^17^. Hall *et al*. integrated RNA-seq, DNAm, and microRNA datasets in islet cells for a sex comparison to identify genes associated with insulin secretion, revealing the potential molecular mechanism for phenotypic sex differences in this tissue^18^. At the DNAm level, we previously focused on chrX to compare between sexes in 27 tissues using Illumina 450 K arrays, from which we identified escapees and subject genes across cell types^19^. The assay of transposase accessible chromatin with sequencing (ATAC-seq) maps chromatin accessibility in a given cell population. A sex comparison on chrX by Qu *et al*. using ATAC-seq data in T cells revealed the highest female to male ratio of chromatin accessibility at *XIST* and *FIRRE*, followed by escapees^20^.

Allelic expression or allelic binding approaches are powerful in delineating activities from either Xi or Xa, although they require cells with XCI of one chrX favored over the other (skewed XCI) as well as the genotype information of the cells. These studies in general are limited by the availability of known heterozygous sites within the particular cells for allelic comparison. Experiments using F1 mouse cells containing large numbers of known heterozygous sites have therefore been favored. In human lymphoblastoid cell lines, allelic expression and histone modifications, measured using Illumina BeadChip genotyping arrays, showed the majority of genes to be subject to XCI, and a correspondence between expression and histone modifications^21^. Allelic RNA-seq and ChIP-seq for RNA polymerase II and various TFs have previously been conducted on the lymphoblastoid GM12878 cell line known to have skewed XCI^22^,^23^,^24^. Reddy *et al*. reported that TFs are predominately bound on the Xa in GM12878, and allelic binding of RNA polymerase II is non-differential between Xa and Xi at escapees^24^.

The recent generation of high throughput datasets offers new opportunities to examine X-linked gene expression. The cap analysis of gene expression (CAGE) datasets, which measure abundance of the 5′ end of transcribed RNAs, generated by the FANTOM5 consortium have facilitated the identification of transcription start sites (TSSs) and their levels of transcription in over 800 samples of non-treated human cells^25^. The strength of CAGE datasets compared to RNA-seq lies in the capability of pinpointing TSSs of gene promoters as well as enhancers^25^,^26^. For the ease of terminology, we refer to the reported robust CAGE peaks as TSSs in the rest of the text. In addition, The Cancer Genome Atlas (TCGA) offers a rich source of DNAm datasets of both sexes in specific cancer types from the epigenetic perspective^27^, while over 680 TF ChIP-seq datasets generated by the ENCODE project provide information on TF binding events in multiple cell types^28^,^29^.

Here we report a comprehensive investigation of X-chromosome gene activity between the sexes, focused on escapees through direct sex comparisons of expression, DNAm, and TF binding levels, further supported with evidence of allelic binding on chrX in the female GM12878 lymphoblastoid cell line. While XCI has previously been reported to occur at a domain level^30^,^31^, we hypothesize that there exists a common regulatory mechanism that facilitates escape from XCI. To our knowledge, this is the first study conducting large-scale analyses on TF ChIP-seq data comparing between sexes and between Xi and Xa occupancies to identify potential regulator(s) associated with escape from XCI.

## Results

### Sex classification of the FANTOM5 samples using CAGE data

**Figure 1:**
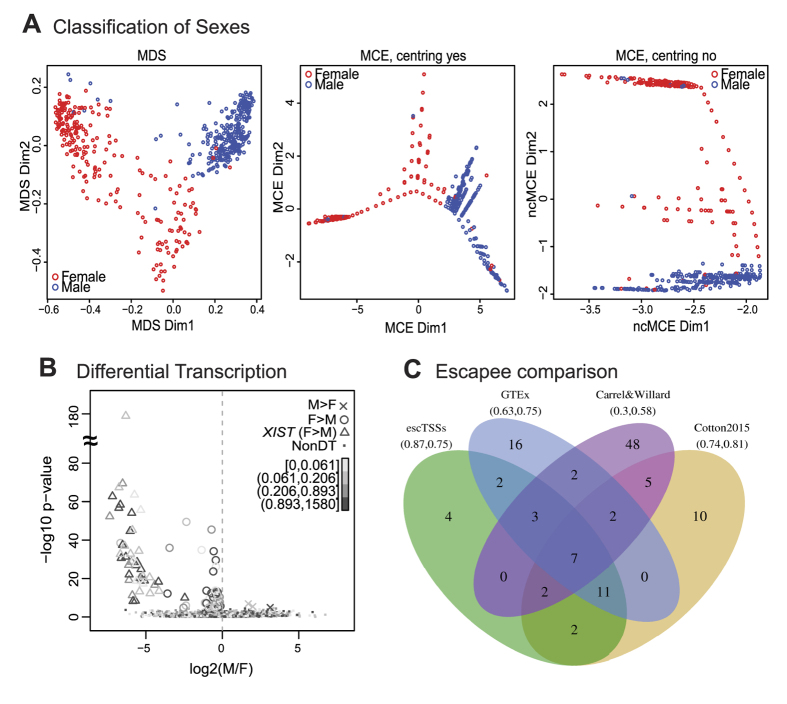
Classification and differential expression analysis of sexes using FANTOM5 CAGE datasets. (**A**) Plots showing distances between FANTOM5 CAGE samples in the first 2 dimensions generated using multi-dimensional scaling, Minimum Curvilinear embedding, and non-centred Minimum Curvilinear embedding methods from the proximity matrix of the Random Forest sex classifier. Each circle represents a FANTOM5 sample with its labeled sex: male (blue) or female (red). (**B**) Scatter plot with log2 ratio of the mean expression levels comparing male to female (with a constant of the 5^th^ percentile expression added to avoid denominators of 0) on the x-axis, and -log10 transformation of raw p-values from the differential transcription analysis between sexes on the y-axis. Each point represents a TSS in non-PAR region of chrX, and TSSs with significantly higher expression in female (escTSSs) and male cells are denoted with circles and crosses (Bonferroni-corrected p-value ≤ 0.05), with small dots for non-differentially transcribed TSSs. The escTSSs nearest to the *XIST* gene are denoted using open triangles. The grey-scale gradient represents the average expression across all samples in quartiles. The vertical dashed line represents a log2 ratio of 0, where there is no difference between sexes. (**C**) Venn diagram depicting the overlapping sets of escapees from three published studies with those identified in this report. The numbers within the Venn diagram represent the overlaps between sets, and the numbers in bracket under each list name are precision and recall values where genes reported in more than one list are taken to constitute true escapees.

To understand how well the transcript levels at TSSs can distinguish sexes and to confirm reliable labeling of male and female samples, we built a sex classifier through a Random Forest approach^32^ using the FANTOM5 CAGE dataset^25^. It is important to note that the datasets contained a mixture of samples from primary cell types, tissues and cell lines, which may have distinct properties. While biological replicates create stronger data consistency, the inconsistent availability of technical replicates creates over-emphasis on those samples with replicates. Therefore only the first replicate from each source was retained. We trained on 530 samples of known sex using the transcript levels of 5,071 TSSs in the non-pseudoautosomal region of the X (X-non-PAR) as features. Through performance assessment, the classifier performed better than a classifier based on TSSs assigned to *XIST* alone. Namely, the out of bag error rate and balanced accuracy were 6.51% and 0.908 for the X-non-PAR classifier and 11.55% and 0.825 for the *XIST* classifier. Hence, we used results from the X-non-PAR classifier in our following analyses. Linear and nonlinear dimension reduction of the sample proximity values from the Random Forest classifier consistently indicated the presence of outlying samples, as shown with multi-dimensional scaling and Minimum Curvilinear embedding^33^ in Fig. 1A. The *XIST* TSSs of these outlying samples were differently expressed from their original sex labels (Supplementary Table S1). Of the 26 samples called female but classified male, there were 2 stem cells and 14 cancer lines, in which the *XIST* expression were zero to minimal. The latter group is consistent with reports of certain cancer cells losing the Xi^34^. Of the 10 samples labeled male but classified female, there were 3 testes-related tumor cell lines with strong *XIST* expression in agreement with a previous report of testicular germ cell tumors expressing *XIST*^35^. The remaining samples could reflect sex chromosome aneuploidy or sample mislabeling. For the latter we noted at least one case where the labeled sex differed between technical replicates from the same source. Overall, the results suggested that we can classify sexes accurately using transcript levels of TSSs in X-non-PAR regions. Thus we constructed our sex classifier using X-non-PAR TSSs training on all labeled samples, and predicted a total of 309 female and 426 male samples to be used in the subsequent section with the exclusion of outlier and mixed samples (Supplementary Table S1).

### Differentially transcribed chrX TSSs with higher expression in female reflect escapees

Given that there were unbalanced numbers of samples between the sexes for multiple cell types (Supplementary Table S2), we incorporated cell categories from ontologies to group multiple closely related cell types to minimize bias from cell-type specific transcription. Using the sex labels from our classifier, we assessed the differential transcription between sexes using a linear regression model for each TSS, incorporating the cell categories as covariates (see Methods for details). Parallel analysis was conducted on both the X-non-PAR region and autosomes (Supplementary Table S3). Here we focused on chrX, with information from the autosome analysis presented in Supplementary File S1. TSSs that overlap repetitive elements were excluded from the analysis. Of the 94 TSSs on chrX that were significantly differentially transcribed between sexes with Bonferroni-corrected p-values < 0.05, two and one TSSs assigned to the *ARHGAP4* and *SH2D1A* genes, respectively, had higher expression in male cells, while 91 TSSs (referred to as ‘escTSSs’) corresponded to 31 unique genes with higher expression in female cells (Fig. 1B). Forty-five of the 91 escTSSs were associated to the *XIST* gene, and exhibited much stronger differences between sexes than others (Fig. 1B).

Given the higher expression in female cells, we expected the majority of our predicted escTSSs to escape from XCI (Supplementary Fig. S1A). Consistent with previously reported escape of an alternative TSS for *UBA1* gene^36^, we identified escTSSs overlapping the alternative TSS while other *UBA1* TSSs were not significantly different between male and female. CAGE advantageously provides precise information about individual TSSs, while other methods are constrained to gene-level resolution. We compared the associated genes of our escTSSs to previously identified escapees from the literature, in which different data and techniques were used: RNA-seq (‘GTEx’)^37^, rodent/human somatic cell hybrids (‘Carrel&Willard’)^38^, and Illumina 450 k DNAm array (‘Cotton2015’)^19^. For our comparison, the escapees identified by at least two approaches were assumed to be true escapees (these are inclusive of reported brain-specific escapees^17^). Our analysis was geared to identify TSSs that broadly escape across cell types. As escape genes can express at different levels from Xi between cell types and samples^19^,^38^,^39^,^40^, the comparison included only genes that broadly escape among samples and tissues, where such information was provided (detailed in Methods). Twenty-seven of the 31 genes from our list were captured by at least one publication, and four genes (five escTSSs in total) appeared to be novel (Fig. 1C). There were 9 genes that were not detected in our set and appeared in two or more published escapee sets, of which 2 occurred in all 3 published sets (*GYG2* and *STS*). Our approach using CAGE data obtained the highest positive predictive value for escapees (precision = 0.87), and ranked second after ‘Cotton2015’ for the proportion of true escapees predicted (recall = 0.75).

We further examined the five apparently novel escTSSs we predicted. Notably, all five escTSSs were on the opposite strand of known escapees identified in two or more studies (Intron 1 of *DDX3X*, Exon 6 of *XIST*, Intron 1 of *HDHD1* and Exon 5 of *MXRA5*). Three novel escTSSs belonged to two non-coding RNAs: *TSIX* and *MIR4767*. The other two novel escTSSs were distant from the nearest annotated genes (11 kb and 44 kb away from *RN7SL15P* and *ASS1P4*, respectively), and therefore it is unclear to which genes they are best ascribed. We successfully validated the escTSS which was antisense to *DDX3X* and had the highest significance in differential transcripts between sexes (Supplementary Fig. S2). *DDX3X* antisense transcripts can be observed from previously published GRO-seq data in IMR90, K562 and GM12878 female cell lines^41^,^42^, and interestingly, our validation with mouse-human hybrid cells indicated that the antisense transcripts were Xi-specific (Supplementary Fig. S2). While the two *MIR4767-*labelled escTSSs had high GC content making them difficult to validate, recently updated RefSeq annotation shows that *MIR4767* overlaps the 5′ end of two *STS* transcripts. Therefore, it may be more accurate to assign the two escTSSs as alternative promoters of *STS* (a known escapee noted above as one of the two undetected genes). Summarizing, CAGE-based detection of escTSSs had the highest precision compared to the published sets.

### DNA methylation similarity between sexes on chrX showed strong agreement with bi-allelically transcribed escapees

**Figure 2:**
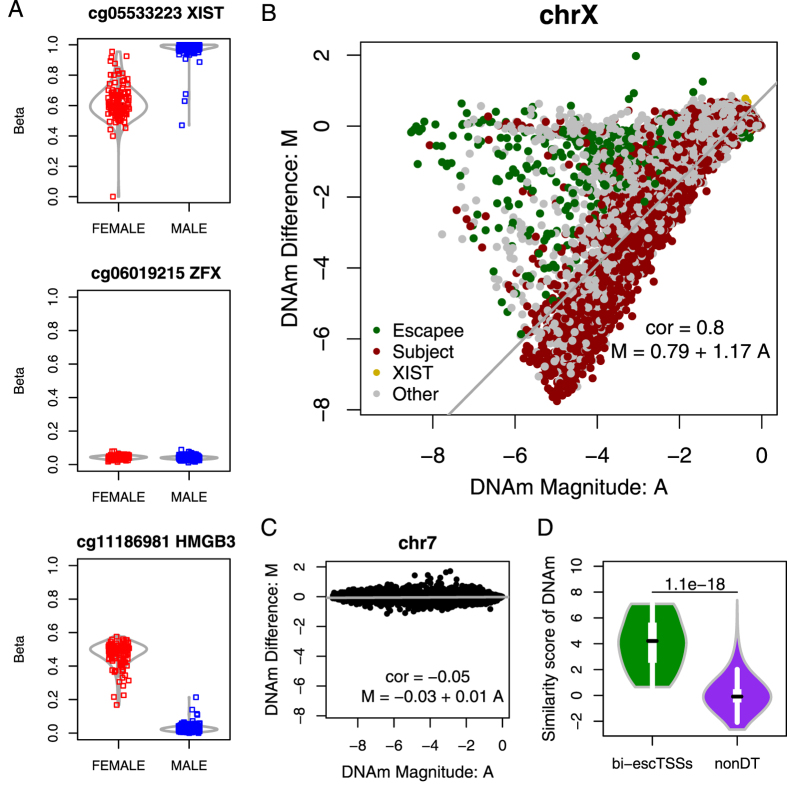
DNAm comparison between sexes on chrX in urothelial bladder cancer (BLCA) samples from TCGA. (**A**) DNA methylation status for positions (i.e. probes from the Illumina 450 k array) near TSSs in both sexes from BLCA samples, where the *β* values (Y-axis) range from 0 (unmethylated) to 1 (fully methylated). The three TSSs are most proximal to the following genes (from top to bottom): *XIST*, an escapee (*ZFX*) and a subject gene (*HMGB3*). Each square represents a sample for the BLCA dataset. Red or blue color represents a female or male sample, respectively. Each violin plot in gray lines shows the distribution of beta values for each sex at each probe. Plots (**B**,**C**) show MA plots for chrX probes and autosomal probes on chr7 between sexes, respectively. Each dot represents a probe from the array. M (difference) on y-axis is the logged differential methylation value between sexes, and A (magnitude) on x-axis is the logged average methylation value (as indicated in Methods). The fitted robust regression line is represented in gray, with the corresponding function and correlation reported. Green and red colors in plot (**B**) represent probes nearest to escapees and subject genes previously reported in Cotton *et al*. 2015. Gold and gray colors represent probes nearest to *XIST* and genes not in either three categories. (**D**) Violin plots showing the distributions of DNA methylation similarity scores between sexes for probes within 50 bps of escTSSs and non-differentially transcribed (nonDT) TSSs on chrX. The similarity score of DNA methylation on y-axis is the residual of M as a function of A on chrX. Only TSSs with at least one probe within 50 bps were plotted, and for those TSSs within 50 bps of multiple probes, the average similarity scores of probes were obtained. The p-value from the Wilcoxon test is reported above the violin plots.

We have previously used DNAm data to identify genes that were subject to or escaping from XCI^19^. Here we used an independent public DNAm dataset from TCGA^27^ to examine whether the differential transcription we identified was reflected at DNAm level between the sexes. On autosomes, we expected differential DNAm between the sexes to correspond to differential transcription. On chrX, however, the overall methylation level was reported to be higher in female than in male due to the nature of the Xi^19^, but we expected similar DNAm levels between sexes at bi-allelically transcribed escTSSs (Supplementary Fig. S1B). For ease of reference, we refer to bi-allelically transcribed escTSSs as ‘bi-escTSSs’ for the rest of the text. DNAm of probes near TSSs of *XIST, ZFX* (a bi-allelically transcribed escapee), and *UPF3B* (a subject gene) are shown as examples in Fig. 2A using data in female and male urothelial bladder cancer samples from TCGA.

To show the differences in DNAm between sexes on chrX and autosomes, we generated microarray-inspired MA plots to compare the relationship between log2 ratios of DNAm between male and female samples (i.e. the difference: M) versus log2 average of DNAm between the sexes (i.e. the magnitude: A) for probes in chrX and chr7 (Fig. 2B,C; see methods for details). We discovered a drastically stronger linear correlation between difference and magnitude on chrX (ρ = 0.8) but not on autosomes (chr7: ρ = −0.05). On autosomes, probes that deviate from the zero difference values were differentially methylated between sexes; whereas on chrX, probes that deviate from the fitted regression line, with the log beta ratio closer to 0, had similar DNAm levels between sexes. Hence, we identified the chrX probes with similar DNAm levels between sexes by computing the residual of the regression model as the similarity score, which also gives greater weights to probes with lower DNAm levels. Indeed, the DNAm similarity scores from probes within 50 base pairs (bps) of TSSs were found to be significantly higher for bi-escTSSs than non-differentially transcribed TSSs (nonDT; p-value of Wilcoxon test: 1.1*10^−18^; Fig. 2D). We observed similar results using three other cancer datasets (Supplementary Fig. S3). Taken together, the results showed that higher DNAm similarity between sexes on chrX reflected TSSs of escapees, and further supported the bi-allelic activities of the bi-escTSSs we predicted.

### YY1 binding motif over-representation around escTSSs

**Figure 3:**
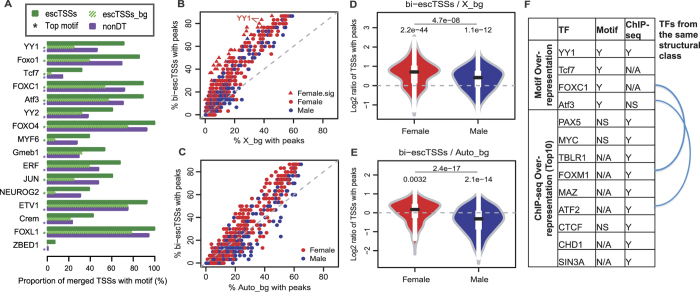
Over-representation analyses of TF binding motifs and ENCODE TF ChIP-seq peaks at escape TSSs. (**A**) Barplots showing the proportions of merged TSS regions (x-axis) containing the JASPAR TF motifs labeled on the y-axis for escTSSs (green) and non-differentially transcribed set on X with Bonferroni-corrected p-values equal to 1 (nonDT; purple). The proportions for %GC composition and length matched background set sampled from the genome for escTSSs (escTSSs_bg) is shown in shaded green. The top motifs with Fisher scores greater than 95^th^ percentile in escTSSs compared to either background set (escTSSs_bg and nonDT) are plotted. The top motifs derived with each background set are marked with asterisks under the background bars with corresponding color. Motifs are presented in decreasing order of Fisher score sums from both comparisons. Figures (**B**,**D**) compare bi-escTSSs to matched background TSSs on chrX (X_bg) for over-representation of TF ChIP-seq peaks, whereas figures (**C**,**E**) compare to matched background TSSs on autosomes (Auto_bg). (**B**,**C**): Scatter plots showing the percentages of TSSs that overlap a peak comparing between bi-escTSSs (y-axis) and matched background TSSs (x-axis). Each dot represents a uniformly processed TF ChIP-seq dataset from ENCODE. Red and blue colors represent female and male cells in all plots, respectively. The dashed gray lines are the baselines reflecting no differences between proportions of escTSSs and background TSSs overlapping peaks. Datasets with significant over-representation of peaks in escTSSs compared to background TSSs are displayed as triangles (Bonferroni-corrected p-values ≤ 0.05). Significantly over-represented YY1 datasets are labeled on figure (**B**). (**D**,**E**) Violin plots showing the distributions of log2 ratio of escTSSs to background TSSs in female and male cells. The p-value from comparing the log2 ratios between male and female cells (one-sided Wilcoxon test) and the p-values of one-sample Wilcoxon tests for the distributions are shown. (**F**) Figure listing TF motifs over-represented compared to both background sets, and the top 10 over-represented TFs in ranking of significance. The semi-circle lines link TFs within the same structural classes. ‘N/A’ indicates that data is unavailable. ’Y’ and ‘NS’ indicate significant or not significant over-representation, respectively.

Given that escTSSs (both bi-escTSSs as well as *XIST*) are transcribed on the Xi, we began testing our hypothesis that common regulator(s) and mechanism exist to facilitate the escape from XCI. We first probed the regulation of the escTSSs on chrX at the sequence level through enrichment testing of JASPAR motifs^43^ using the CAGEd-oPOSSUM web tool^44^. Merged sequences from 500 bps up- and down-stream of escTSSs were compared to two background sets: nonDT and a randomly selected %GC and length matched background set (escTSSs_bg). The TF binding motifs associated with YY1, Tcf7, FOXC1 and Atf3 were found to be over-represented around escTSSs when compared to both background sets (Fig. 3A and Supplementary Table S4). The significance of YY1 binding motif was highest among all motifs, and it was 2.9 and 1.5 times more frequently found in escTSSs than escTSSs_bg and nonDT sets, respectively. The motif over-representation analyses indicated YY1 as a potential regulator of escapees through sequence specific binding.

### Over-representation of TF ChIP-seq peaks around bi-allelically transcribed escTSSs

To explore the regulation of escapees at the experimental level, we examined TF ChIP-seq data in male and female cell lines from the ENCODE project to identify over-represented TF binding at the bi-escTSSs. TF binding on chrX in female cells can be either bi- or mono-allelic, and the measured binding degree would reflect a mixture of both X chromosomes. Therefore, given a positive-regulating TF, we expect a stronger binding pattern at bi-escTSSs than mono-allelically active TSSs on chrX in female cells (Supplementary Fig. S1C). We refer to this expectation as the bi-allelic effect. As there is only one copy of the chrX in the male cells, enrichment is not expected.

We tested the over-representation of all 689 uniformly processed ENCODE TF ChIP-seq peak sets within 500 bps of bi-escTSSs compared to background TSSs on chrX (X_bg) and autosomes (Auto_bg) with matched average expression (see Methods for details). Thirty individual ChIP-seq datasets, corresponding to 18 unique TFs, were over-represented around bi-escTSSs when compared to X_bg (One-sided Fisher’s exact test, Bonferroni-corrected p-values ≤ 0.05; Supplementary Table S5 and Fig. 3B). The top 10 unique over-represented TFs in the order of decreasing significance were PAX5, MYC, TBLR1, FOXM1, YY1, MAZ, ATF2, CTCF, CHD1 and SIN3A. Notably, the significant TF datasets were all generated from female cells except for YY1 data in the male HepG2 cell line. By contrast, when background was changed to the autosomes (Auto_bg), none of the TF ChIP-seq peaks were found to be significantly over-represented (Supplementary Table S5 and Fig. 3C).

Both motif and ChIP-seq analyses revealed YY1 to be over-represented around escTSSs. For a subset of the top escTSSs (+/−500 bp regions), visual inspection affirms a strong consistency between predicted YY1 motifs and ChIP-seq peaks (Supplementary Fig. S4). We sought to determine if there were additional cases of TFs, which were consistent between both analyses (Fig. 3F). TFs within the same structural class often have highly similar binding properties. For two cases, a motif was enriched for a TF (FOXC1 and Atf3), and ChIP-seq was enriched for another member of the same structural class (FOXM1 and ATF2, respectively). Four of the top 10 TFs with ChIP-seq over-representation lacked binding models. ChIP-seq peak but not motif over-representation of PAX5, c-Myc and CTCF might reflect a presence of these TFs via secondary mechanisms (not sequence-specific DNA binding). As an independent validation, we examined two published X;autosomal translocation studies^30^,^45^, which reported in aggregate 1106 and 473 autosomal genes that escaped from or were subject to the spread of heterochromatin from Xi in trisomic cells, respectively. When comparing autosomal escapees to autosomal subject genes, we found YY1 and MYC (ranked 6^th^ and 8^th^) to be among the top 10 unique TFs with ChIP-seq peaks over-represented around escapee TSSs (Supplementary Table S6). Taken together, the significance of YY1 binding in multiple cellular contexts from the chrX and X;A data further highlighted the strong potential for a role for YY1 in facilitating the escape from XCI.

While the 30 ChIP-seq datasets have peaks significantly over-represented at escTSSs compared to X_bg, many additional sets (250 of 689) were reported non-significant after the conservative Bonferroni correction was applied. We observed an overall trend of higher proportions of escTSSs containing peaks compared to that of X_bg (Fig. 3B). Indeed, the distribution of log2 ratios of percent overlaps with TF peaks (escTSSs/X_bg) in both female and male cells were significantly greater than 0 (one-sample Wilcoxon test p = 2.2*10^−44^ and 1.1*10^−12^, respectively; Fig. 3D). In agreement with our expectation, the log2 ratios in female cells were significantly higher than that observed in male cells (one-sided Wilcoxon test p = 4.7*10^−08^). The log2 ratios comparing escTSSs to autosomal background (Auto_bg) was less significantly different from 0 in female cells (p = 0.0032; Fig. 3E), whereas it was significantly lower in male cells (p = 2.1*10^−14^), due to the single copy of chrX in male cells. The overall higher number of binding events at escTSSs stressed the importance of taking multiple TF datasets into consideration when comparing between sexes at escTSSs or between escTSSs and other chrX TSSs, otherwise the differential occupancy may merely reflect the bi-allelic effect.

### ChIP-seq read depth reveals overall reduction of input from heterochromatic Xi

**Figure 4:**
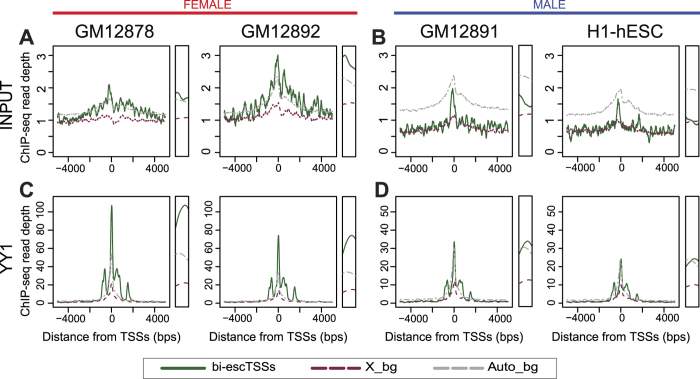
Input and YY1 ChIP-seq read depths around bi-escTSSs for both sexes. The read depth plots for ENCODE ChIP-seq input samples in two female (**A**) and male cell lines (**B**) within 5 kb of three TSS sets: a subset of bi-escTSSs in green with a filter of unique escTSS per gene symbol, background TSSs on chrX with matched averaged expression (X_bg) in violet dashed line, and autosomal TSSs with matched averaged expression (Auto_bg) in gray dashed line. The read depth plots for ENCODE YY1 ChIP-seq data in the same female (**C**) and male cells (**D**). The scales on y-axis of figures A and B are the same, while the scale of C is two times the scale of D to reflect the expected X-copies in female and male cells. For the ease of visualization, the narrow panel on the right of each plot displays the read depth within 50 bps of the TSSs.

As TF binding peaks do not necessary reflect linearly the magnitude of TF occupancy, we further compared the read depth at escTSSs to the same background sets using input and YY1 ChIP-seq data from two female and male cells. Although female cells have two copies of autosomes and chrX, the read depth for input within ±50 bps of X_bg was 0.68 fold lower than that of Auto_bg on average, while the read depth within ±50 bps of escTSSs was closer to Auto_bg (1.18 fold; Fig. 4A). This indicated a discounted number of input fragments available for capture due to the compactness of Xi around X_bg in female cells. While male cells have two copies of each autosome and one of chrX, the input read depths at chrX (X_bg and escTSSs) and autosomes (Auto_bg) reflected the ratio as expected (0.50 and 0.58 fold, respectively; Fig. 4B). Overall, the input reads in female cells reflect bi- and mono-allelic activities at escTSSs and X_bg, respectively, and input reads at X_bg in female cells, while somewhat higher than those in male cells, reflect a reduction of the heterochromatin input.

### YY1 binding at escTSSs shown by read depth and allelic analyses

Interestingly, despite slightly lower input levels, YY1 ChIP-seq read depth at escTSSs was 2.01 times that of Auto_bg in female cells (Fig. 4C). Furthermore, despite the 1 to 2 ratio of input levels at escTSSs and Auto_bg in male cells, the YY1 read depths at escTSSs and Auto_bg were similar (1.05 fold; Fig. 4D). In contrast, the YY1 read depth ratio between X_bg and Auto_bg was similar to that of input read depth in both female and male cells (Fig. 4C,D). Overall the results showed higher occupancy of YY1 at escTSSs compared to both X_bg and Auto_bg for both sexes, and thus enhanced YY1 binding may predispose to ongoing transcription from the inactive X.

Given that YY1 motifs and ChIP-seq peaks were over-represented at escTSSs, we next examined the allelic binding of YY1 on chrX in the female GM12878 cell line to probe its functional role, and confirm its bi-allelic binding at bi-escTSSs (see also schematic in Supplementary Fig. S1D). We extracted the YY1 ChIP-seq read counts on Xi and Xa at the 67 heterozygous sites within YY1 ChIP-seq peaks in GM12878 (Supplementary Table S7). Only two YY1 peaks containing heterozygous sites were within 500 bps to two bi-escTSSs. Indeed, YY1 was bound on both Xa and Xi at both sites, within exon 1 of *SMC1A* (7:13, representing counts of Xa:Xi) and exon 1 or exon 2 of *JPX* transcripts (12:8), confirming bi-allelic transcription and bi-allelic YY1 binding of the escapees.

Consistent with previous reports of Xa-biased binding of TFs reflecting Xa-biased transcription in female cells, the majority of heterozygous sites (52 out of 67) had more reads on Xa than Xi, while 13 heterozygous sites had more reads on Xi. Despite the overall trend of Xa-biased binding, we found the highest number of allelic YY1 reads on Xi (3:474) within exon 1 of *XIST.* The second highest number of YY1 reads was at 215 bps upstream of *UPF3B* (140:0), and the Xa-biased binding agreed with its subject status reported previously^19^. The site with the third highest number of YY1 reads overlapped intron 5 of *FIRRE* and was Xi-biased (4:138). Interestingly, Xa-biased YY1 was detected at sites 477 bps upstream of *FIRRE* TSS and in intron 1 (44:5 and 20:0). Such discrepancy was consistent with the previous report of a shorter alternative *FIRRE* transcript on Xi^9^ and the finding of female-specific enhancers in introns 2–12 from ATAC-seq^20^. The Xa- or Xi- specific binding of YY1 might reflect its reported capacity for methylation-sensitive binding^46^, and/or be due to cofactors acting at escapees in collaboration with YY1. Overall, the allelic binding reads of YY1 indicated a positive association with regulation of X-linked genes.

### Significant Xi-biased YY1 occupancy at *XIST, FIRRE* and two other superloop-associated lncRNAs in GM12878

**Figure 5:**
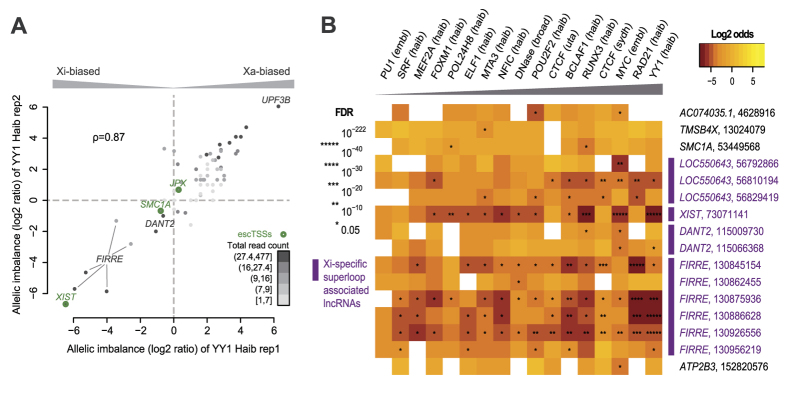
Allelic imbalance at heterozygous sites within ChIP-seq peaks on chrX in the GM12878 cell line. (**A**) Scatterplot showing the allelic imbalance of replicated YY1 ChIP-seq data sets from ENCODE (see Methods). For visualization purposes, allelic imbalance is represented by the log2 ratio of (Xa + 1) to (Xi + 1). A positive log2 ratio value indicates more reads on Xa, while a negative value represents more reads on Xi. Each of the 67 dots represents a heterozygous site within a YY1 binding peak. The Pearson correlation between allelic imbalance of the replicated datasets is 0.87. Dotted lines indicate the baselines for balanced allelic binding. Heterozygous sites within 50 bps of escTSSs are indicated by green circles. The intensity of shading of each dot reflects the total number of YY1 reads from both replicates at the heterozygous site (where read counts were assigned to five 20 percentile bins). (**B**) Heatmap showing heterozygous sites (rows) significantly Xi-biased in more than one dataset (ChIP-seq and DNase I data; column). Only datasets that are significantly Xi-biased at more than four heterozygous sites are listed. Datasets are denoted by the feature name followed by the ENCODE lab where data was generated, and heterozygous sites are denoted by the gene name of the nearest TSS followed by the chrX coordinate of the site. Colors in the heatmap represent log2 odds ratio values reflecting Xa- or Xi-biased binding of the TF with positive (gold) or negative (brown) values, respectively. The log2 odds ratio distinguishes Xi bias (negative) and Xa bias (positive). White boxes indicate zero read counts at the corresponding site-data pair. The degrees of significance estimated by FDR-corrected p-values are indicated on a scale of 1 to 5 asterisks with the corresponding p-value thresholds shown in the legend. The datasets from left to right are ordered in increasing counts of higher significance scales denoted by the gray triangle, and the heterozygous sites are ordered using genomic coordinates on chrX. The four lncRNAs previously reported to be associated with Xi-specific superloops are marked with purple bars and colored in purple.

Given the important roles of *XIST* and *FIRRE* on XCI and the strong allelic imbalance of YY1 binding at these lncRNAs, log2 ratios of Xa to Xi counts were computed to identify strong allelic imbalance at all 67 heterozygous sites (see Methods for detail). The significant correlation between allelic imbalance scores from replicates of YY1 ChIP-seq datasets indicated strong consistency (ρ = 0.87 with p < 2.2*10^−16^; Fig. 5A). Heterozygous sites at exon 1 of *XIST* and intron 5 of *FIRRE* were shown to have the strongest Xi-biased YY1 binding. These sites with YY1 binding were found to be top 5 in significance of allelic imbalance when we extended the allelic analysis to all available ChIP-seq and DNase I datasets in GM12878 as well as 1,321 heterozygous sites, and tested each heterozygous site and data pairs for imbalance significance (see Methods for details and Supplementary Table S8 for raw read counts). We identified 389 significant imbalance site-data pairs comparing to the overall Xa-biased norm using False Discovery Rate (FDR) correction (discussed in Supplementary File S1, and Fig. S5), which corresponded to 178 unique heterozygous sites (FDR corrected p-values ≤ 0.05; Supplementary Table S9). Thirty-eight out of 178 sites exhibited imbalance in more than one dataset, within which 16 and 18 sites were biased consistently across data sets towards Xi and Xa, respectively. Such consistency in allelic imbalance across TFs indicated broader influence such as open or closed chromatin rather than individual TF binding affinity of alleles.

Interestingly, the majority of significantly Xi-biased imbalances mapped to the vicinity of the four lncRNAs associated with Xi-specific superloops (Fig. 5B and Supplementary Table S9). This finding is complementary and consistent to previous reports on Xi-specific binding of YY1 and CTCF to two of the four lncRNAs, *DXZ4* and *FIRRE,* in mouse and human^9^,^47^,^48^,^49^. YY1 binding at the heterozygous site within *XIST* was the most significantly Xi-biased across all data sets and sites (FDR-corrected p-value = 7.12*10^−221^), while 21 other datasets were also significantly Xi-biased at the same site to a lesser degree. The Xi-biased YY1 binding at heterozygous sites around intron 5 of *FIRRE* were ranked fourth and fifth in overall significance, whereas RAD21 and CTCF were preferentially Xi-bound with lower ranks. While no heterozygous sites overlapped *DXZ4*, significant Xi-biased sites overlapped the *DXZ4* associated non-coding transcript 2, *DANT2*. Notably, *DANT2* is located within the previously reported boundary of two Xi superdomains, which were not associated with Xa or in male cells^14^. Overall, significant Xi-biased bindings of YY1 and MYC were found in all four lncRNAs, while RAD21 and CTCF were significantly Xi-biased at *FIRRE* and *LOC550643*, but not *XIST* and *DANT2*. The allelic analysis revealed Xi binding of YY1 at Xi-transcribed genes in general, including escTSSs and key lncRNAs involved with XCI, Xi-specific superloops and the superdomain boundary.

## Discussion

Here we analyzed CAGE datasets reporting the transcription levels at chrX TSSs to identify escTSSs through differential transcription analysis between male and female samples matched for cell categories. Predicted escTSSs yielded the highest precision and the second highest recall for escapee gene identification compared to three reports using different techniques. In addition, we experimentally validated a novel escapee, which was antisense to an escapee, *DDX3X*. Analyses of experimental data revealed unique properties of escapees and the resulting bias of bi-allelic activity in data. Over-representation analyses on motifs and ChIP-seq data suggested involvement of YY1 sequence-specific binding at escTSSs. Consistently in an independent context (X;autosomal translocation study), over-representation of YY1 ChIP-seq peaks around autosomal escapee TSSs supported the link between YY1 and escapees. Allelic binding analysis in GM12878 cells further indicated a role for YY1 proximal to lncRNAs involved with Xi-specific superloops. In aggregate, the analyses showed that YY1 is associated with Xi expression of not only *XIST* but more broadly of escapees.

From a bioinformatics perspective, the analysis of chrX is challenging. In female datasets, measurements captured are a combination of properties from Xa and Xi. Genes subject to XCI are under a mixture of positive (Xa) and negative (Xi) regulation, whereas bi-allelically transcribed escapees are positively regulated on both X chromosomes. Direct use of tools and approaches designed for autosomes can confound interpretation. Our analyses explored properties of bi-escTSSs both in DNAm and TF binding perspectives. Despite having higher expression in females, bi-escTSSs have similar total DNAm levels between sexes consistent with bi-allelic activity in females. In ChIP-seq datasets, there are more bi-escTSSs with peaks than the mono-allelically active background TSSs on chrX for both sexes (recall Fig. 3D). Such over-representation in female datasets resulted from a combination of the bi-allelic effect, and predispositions of TFs to bind at escapees. We have revealed properties of escapees by addressing chrX with targeted analyses of genome-scale data.

YY1 was previously implicated to trans-activate *XIST* expression through tethering *XIST* RNA^12^,^13^, and knocking-down YY1 significantly affected *XIST* expression^50^. Complementary to previous studies, our report of motif and ChIP-seq peak enrichment at bi-escTSSs and allelic binding suggested that YY1 plays a broader and positive role in the regulation of escapees through sequence-specific binding. Furthermore, adding to previous reports of Xi-specific binding of YY1 and CTCF at *DXZ4* and *FIRRE*^9^,^47^,^48^,^49^, our analysis identified significant Xi-specific binding of YY1 and MYC at all four lncRNAs frequently associated with superloops in GM12878 cells. A plausible mechanism for the observed YY1 allelic binding preference toward Xi is its binding sensitivity to DNAm^12^,^46^.

Combining our results with the literature, we postulate two non-exclusive mechanistic models for YY1’s involvement in the regulation of escapees: trapping by RNA and chromatin looping. A RNA trapping model is based in part on the capability of YY1 to bind both DNA and RNA^11^,^28^,^51^, localizing *XIST* RNA to the X nucleation center^11^. Supporting the trapping model, a recent report showed enhanced YY1 occupancy on DNA leads to higher stability in gene expression when YY1 interacts with nearby RNAs^51^. Alternatively, the chromatin looping model is supported by the observation that YY1 participates in controlling long distance DNA interactions during B cell development^52^,^53^, and the enrichment of YY1 ChIP-seq peaks at boundaries of large chromatin domains^54^. As active regions on Xi loop away from the condensed Xi^55^,^56^,^57^, the significant YY1 Xi-biased binding at the four lncRNA loci, where Xi-specific superloops are highly interactive, indicated a potential involvement of YY1 to chromatin looping.

Overall the results support a model in which YY1 facilitates escape from XCI, but the analyses are generally limited by the availability of data. At the transcript level, our approach in identifying differentially transcribed TSSs from FANTOM5 CAGE datasets was limited to broadly escape genes, as there are few precisely matched male and female samples. Variability of escape from XCI between tissues has been observed^19^,^38^,^39^,^40^, therefore the role and the regulation of tissue-specific escapees are important future directions. Further investigation of relationships between cell categories and sexes could enable the identification of cell type-specific escapees, but will require sufficient numbers of samples from both sexes in the same cell type. At TF binding level, both the JASPAR motif collection and the ENCODE ChIP-seq datasets are far from complete, covering only a subset of TFs. Furthermore, the allelic imbalance analysis in human is particularly constrained to a small set of heterozygous sites within TF peaks and within the female cell lines that are skewed in XCI. Lastly, our allelic analysis on chrX did not take into account the TF binding affinities towards different alleles, as we intended to capture the epigenetic influence from XCI, which was supported by the observation of multiple TFs sharing significant Xi-biased binding at individual sites. Despite the limitations, we were able to reveal the intrinsic data properties of X-linked genes in males and females, which in turn allowed the identification of the potential regulator of escapees, YY1, and suggested its functional role on the inactive X.

## Methods

All analyses were conducted in R (3.0.2)^58^ and Bioconductor (2.13)^59^ unless otherwise stated.

### Public datasets

For expression analysis and sex classification, the RLE-normalized expression table of robust CAGE peaks for human samples was retrieved from the FANTOM5 consortium^25^. For the DNA methylation analysis, raw data files in idat format from Illumina 450 k array generated by TCGA were retrieved^27^, and the following datasets for four cancer types were used: Bladder Urothelial Cancer (BLCA), Colon Adenocarcinoma (COAD), Head and Neck Squamous Cell Carcinoma (HNSC) and Lung Adenocarcinoma (LUAD). For the ChIP-seq and DNase I analysis, uniformly processed ChIP-seq peaks and DNase I peaks were retrieved from the ENCODE^28^ page on UCSC genome browser^29^. For generating read depth plots, normalized bigwig files from ENCODE were retrieved from https://sites.google.com/site/anshulkundaje/projects/wiggler. For allelic binding analysis, a total of 213 files of ChIP-seq, input and DNase I data from GM12878 cells were retrieved from the ENCODE^28^ page on UCSC genome browser^29^ and from Kilpinen *et al*.^60^. The ChIP-seq peaks of non-ENCODE data were called using MACS^61^ with default parameters.

### Classification and differential expression of the sexes using FANTOM5 CAGE data

All CAGE TSSs were labeled with the gene name corresponding to the nearest TSSs of Ensembl transcripts from ENSEMBL GENES 75^62^. A supervised Random Forest classifier using randomForest R package^63^ was implemented to classify the sexes for the FANTOM5 CAGE data sets. Data from samples involving treatments of the cells or tissues and multiple donations except the first from the same individual were excluded, resulting in a total of 809 datasets. Of these, 296 male and 234 female labeled samples were used as training data. Two classifiers were constructed, one using 5071 TSSs in the non-PAR portions of chrX as features, and the other using the 56 *XIST*-associated TSSs. Performance of the classifier was assessed using 10-fold cross validation as well as out of bag error estimates. For the selected 5,071 TSS-based classifier, sample outliers were identified based on out-of-bag error estimation. The classifier was used to predict samples labeled unknown.

Ontologies associated with the FANTOM5 samples were retrieved from FANTOM5, including cell types (CL), anatomical systems (Uberon), and diseases (DOID)^64^,^65^. One hundred and fifty-three cell category terms with association to at least 30 samples were extracted. To take cell categories into account when assessing differential transcription between sexes, the first 29 principal components of cell categories explaining over 90% variance in addition to sexes were used as covariates of the linear regression model: 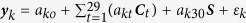, where ***y***_*k*_ is the vector of log10 (tags per million counts at TSS *k* + 5) values in all samples reflecting the level of transcription, ***C***_*t*_ is the vector of rotated cell category relevance values of each sample for the *t*^th^ principal component, and ***S*** is the vector of sexes of samples from the sex classifier. The fitted coefficient of the sex variable, *a*_*k*30_, and its p-value were used to assess differential transcription between sexes of TSS *k*. To reduce the multiple hypothesis testing penalty and avoid bias from non-unique alignment to the genome, 4045 X-non-PAR TSSs that do not overlap repetitive elements were retained for analysis. TSSs with Bonferroni-corrected p-values less or equal to 0.05, and negative *a*_*k*30_ values (higher expression in female samples) were defined as escTSSs.

The precision and recall values compared to escapee lists from the literature were computed using the count of true predicted escapees over the count of all predicted escapees, and the true escapee count, respectively. Genes reported in more than one list were taken to constitute true escapees. Ubiquitous escapees with respect to the samples conducted in the literature were used for comparison: escaping “9/9”, “8/9” in ‘Carrel&Willard’^38^, and “escape from XCI in 27 tissues” in ‘Cotton2015’^19^. Figure 1C is generated using the VennDiagram R package^66^.

### Experimental validation of escape from XCI

Cell culture: The somatic mouse-human hybrid fibroblast cell lines AHA-11aB1, t60-12 (Xa), t11-4Aaz5, t75-2maz34-4a and t86-B1maz1b-3a (Xi) along with the mouse line tsA1S9-az31b were obtained from the Willard lab^67^. Cells were grown in MEM media (Gibco) supplemented with 7.5% Fetal Bovine Serum, L-glutamine and penicillin/streptomycin at 37 °C, 5% CO_2_. Cells were seeded in 100 mm dishes and grown to confluence, harvested using trypsin and stored at −70 °C until RNA extraction.

RNA extraction: RNA was extracted using Trizol (Invitrogen) as per the manufacturer’s instructions. DNA contamination was removed using DNase I recombinant (Roche) as per the manufacturer’s instructions.

Quantitative strand-specific reverse transcription and PCR: The strand specific reverse transcription protocol was performed according to Chapman, *et al*.^12^. Briefly, RNA was reverse transcribed individually for each assay as follows: 13 μL of RNA was incubated with 1 μL 0.50 μM dNTPs and 2 pmol of T7-tagged strand-specific primers at 70 °C for 5 min, then placed on ice for 1 min. Superscript IV buffer (ThermoFisher) was then added to a concentration of 1x, along with 0.005 mol DTT, 1 μL of RiboLock RNase Inhibitor (ThermoFisher) and 1 μL of SuperScript IV enzyme (ThermoFisher), all to a volume of 20 μL. The mixture was incubated at 55 °C for one hour then heat inactivated at 95 °C for 5 min. Reverse transcription for the antisense of *DDX3X* and *ACTIN* were performed independently.

Quantitative PCR (qPCR) was performed by adding 1.5 μL cDNA to 0.2 mM dNTPs, 2.5 mM MgCl2, 1x Hotstart reaction buffer, 1x EvaGreen dye (Biotum), 0.2 μM each of T7 and amplification primer, and 0.8 U Maxima Hot Start Taq (Fermentas). The qPCR was run on a StepOnePlusTM Real-Time PCR System (Applied Biosystems) for 95 °C for 5 min followed by 40 cycles of 20 s at 95 °C, 20 s at 59 °C and 20 s at 72 °C. A melt curve from 52 °C to 95 °C was run to ensure only one product was being amplified. All qPCR runs were done in triplicate. A primer negative control with no primer during the reverse transcription step failed to amplify during qPCR, demonstrating specificity of the qPCR.

### Similarity and differential analysis of DNA methylation between sexes

Due to the complication of XCI in female samples, a basic preprocessing approach using background subtraction of negative control probes and internal control normalization was conducted on all Illumina 450 k DNA methylation data from TCGA using the Minfi R package^68^. The beta value, *β*_*i*_, representing the degree of DNAm was computed for each probe *i* as *Meth*_*i*_/(*Meth*_*i*_ + *Unmeth*_*i*_ + 100) by default. We adopted the analysis of microarray studies by computing the logged differential methylation value between sexes (*M*) and the logged average methylation value (*A*) for each probe *i*: 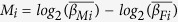 and 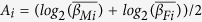, where 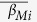 and 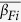 were the average *β* values of probe *i* for the male and female samples, respectively. For probes on chrX, we fitted a linear model, ***M*** = *d*_0_ + *d*_1_***A***, by robust regression using the estimating function of Tukey’s bisquare, which gave extreme observations zero weights. We then obtained the residual of each probe to be the DNAm similarity score between sexes. For assessing the overall correlation between DNAm similarity scores and differential transcription of CAGE TSSs, we assigned probes within 50 bps to each TSS. When multiple DNAm probes are within 50 bps of a TSS, the average of the similarity scores was computed. For probes on autosomes, differential methylation analysis was performed, including age and cancer stage as covariates (adapted from the dmpFinder method of Minfi). F statistics were computed by comparing the goodness of fit of the models using age and cancer stage as covariates with and without the addition of sex variable. Bonferroni-corrected p-values were obtained for each autosomal probe.

### Motif over-representation tests of escTSSs using CAGEd-oPOSSUM

The escTSSs were subjected to motif over-representation analysis using our CAGEd-oPOSSUM web tool^44^. A total of 478 JASPAR2016 motifs with a minimum specificity of 8 bits for vertebrates^69^ were used for the prediction of binding sites. The “Use only FANTOM5 CAGE peaks identified as true TSSs by the TSS classifier” option was selected to filter out CAGE peaks that were less likely to be TSSs. The remaining CAGE peaks were extended 500 bps up- and down-stream, and merged. As background property matching is important for motif over-representation analyses to avoid biases, two tests were conducted differing in background sets: (i) random CAGE TSSs with %GC composition and length matched to escTSSs sampled by CAGEd-oPOSSUM, and (ii) 4,939 non-differentially transcribed chrX TSSs with Bonferroni-corrected p-values of 1 from the differential transcription analysis. The TF binding score threshold was set to 85% by default, and the Fisher scores for both tests were retrieved to assess the over-representation significance of each motif around escTSSs.

### TF ChIP-seq peak over-representation testing and read depth plots

Over-representation testing of TF ChIP-seq peaks within 500 bps of bi-escTSSs was conducted using the one-sided Fisher’s exact test. To avoid double counting, only one TSS with the strongest differential expression between sexes was selected per escape gene. As a result, we conducted the analysis on 30 bi-escTSSs, and randomly selected 300 background TSSs from chromosome X (X_bg) and 3,000 from autosomes (Auto_bg) with matched average transcription levels. An additional criterion of Bonferroni p-value equal to 1 (from differential transcription analysis) was used for the X_bg set to avoid selecting borderline escapees. We assigned all CAGE TSSs to 5 equally sized bins according to average expression percentile ranks. The percentage of bi-escTSSs assigned to each bin was determined and a background set was selected to match the distribution across bins. We extracted the counts of bi-escTSSs, X_bg and Auto_bg sets having at least one peak within 500 bps for each ChIP-seq data. Given the overlapping and non-overlapping counts of bi-escTSSs and background TSSs, one-sided Fisher’s exact tests were conducted to test for positive association between the peaks of each ChIP-seq data and the bi-escTSSs. The significance was adjusted for multiple testing using Bonferroni correction. The log2 ratios of bi-escTSSs to each background set with peaks were computed as log2(% bi-escTSSs with peaks/% background with peaks). For the read depth plots, Bwtool^70^ was used to extract the YY1 ChIP-seq and input read depth within 5 kb of the three TSS sets from big wig files (bi-escTSSs, X_bg and Auto_bg). Read depth ratio between any two TSS sets were computed by comparing the average read depths within ±50 bps of the TSSs. The ratios from cells of the same sex were averaged.

### Allelic ChIP-seq and DNase I data analysis of the female GM12878 cell line

Our in-house allelic binding pipeline was used to extract reads at heterozygous sites from GM12878 datasets and assess mapability for filtering^71^. We obtained the genotype data of GM12878 from the 1000 Genomes Project^72^, and a personalized hg19 genome for GM12878 was built by representing single nucleotide variations as degenerate IUPAC codes (eg. Heterozygous alleles G and A are represented by ‘R’). The IUPAC codes allow an equal alignment of reads from both alleles at each heterozygous site without considering the non-reference allele(s) as a mismatch. Raw reads of 214 ChIP-seq, input and DNase I datasets were aligned to this personalized genome, using Novoalign (version 3.01.00: http://www.novocraft.com) with default parameters. The phased information of GM12878 was obtained from Illumina (http://www.illumina.com/platinumgenomes/). As allelic mapping bias may exist at certain locations within the personalized genome, a set of simulated reads were generated for mapability assessment. We first merged all GM12878 TF ChIP-seq binding peaks plus 100 bps flanking regions, and then simulated all the possible 36-bp reads overlapping with the heterozygous sites for each allele and each strand. After mapping the simulated reads to the personalized genome, only 1,321 out of 1,497 heterozygous sites with balanced simulated read counts between two alleles, calculated by requiring the read count of either allele divided by the sum of two alleles to be between 0.6 and 0.4, were kept for further analysis. As paternal X chromosome corresponds to the Xi in GM12878, we assigned the allelic read counts to Xi or Xa at each heterozygous site accordingly.

All replicated data sets generated by the same lab were merged through summing Xi and Xa reads separately at each site, resulting in 101 merged data sets. The only exception was for Fig. 5A, where replicated YY1 datasets generated by the HudsonAlpha Institute for Biotechnology lab (Haib) were displayed separately to show the correlation of allelic imbalance between replicates. For the individual allelic YY1 binding analysis, only 67 heterozygous sites within uniformly processed YY1 ChIP-seq peaks were examined to avoid noise from low occupancy. Allelic imbalance score at site *h* was computed for visualization purposes using the log2 ratio of reads at Xa over Xi with a constant of 1 added to avoid zero denominators: 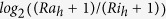, where *Ra*_*h*_ and *Ri*_*h*_ represented read counts on Xa and Xi at site *h*, respectively.

For the batch analysis of allelic imbalance, all 1321 sites were used. The significance of allelic imbalance was assessed using Fisher’s exact test comparing the Xa and Xi counts at each heterozygous site to the total Xa and Xi counts within the corresponding data. For each data *g* at site *h*, the 4 values in 2 × 2 table were *Ra*_*h*_, *Ri*_*h*_, 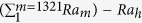, 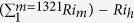, where *Ra*_*h*_ and *Ri*_*h*_ represented read counts on Xa and Xi at site *h* from data *g*, respectively. The significance reflected the extremity of imbalanced site-data pairs compared to the overall Xa-biased total read counts. With the 1321 sites and 101 merged data sets, a total of 61,470 site-data pairs with non-zero read counts from both Xi and Xa were tested. FDR p-value correction was used here because we expected multiple hits of the same TF to multiple heterozygous sites overlapping the same gene. The log2 odds ratio was computed to reflect the direction of imbalance bias with a positive or negative value reflecting bias towards Xa or Xi, respectively: 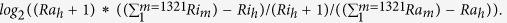


## Additional Information

**How to cite this article**: Chen, C.- *et al*. YY1 binding association with sex-biased transcription revealed through X-linked transcript levels and allelic binding analyses. *Sci. Rep.* **6**, 37324; doi: 10.1038/srep37324 (2016).

**Publisher's note**: Springer Nature remains neutral with regard to jurisdictional claims in published maps and institutional affiliations.

## Supplementary Material

Supplementary Information

Supplementary Dataset 1
