# Supplementary material for: YY1 binding association with sex-biased transcription revealed through X-linked transcript levels and allelic binding analyses
Source: Sci Rep. 2016 Nov 18;6:37324. doi: 10.1038/srep37324 (PMC5114649; doi:10.1038/srep37324)

Supplementary Information

YY1 binding association with sex-biased transcription revealed through X-linked transcript levels and allelic binding analyses

Chih-yu Chen[1,2], Wenqiang Shi[1,2], Bradley Balaton[6], Allison M. Matthews[1], Yifeng Li[1], David J. Arenillas[1], Anthony Mathelier[1], Masayoshi Itoh[3,4,5], Hideya Kawaji[3,4,5], Timo Lassmann[3,4], Yoshihide Hayashizaki[3,5], Piero Carninci[3,4], Alistair R. R. Forrest[3,4], Carolyn J. Brown[6*], Wyeth W. Wasserman[1,6*]

S1 Table. Sex labeling from the Random Forest classification and the list of outliers.
Table A and B list the classified sex from labelled and unlabelled samples, respectively. In Table A, additional information about outlier samples is provided. These entries are distinguished by the column 'Label==pred' being set to FALSE. The 'Female.votes' and 'Male.votes' columns show the proportion of support for each sex within the Random Forest classification process. As *XIST* expression is particularly important for sex classification, the 'sumExpXIST' column reports the sum of normalized tags per million of all TSSs nearest to *XIST* from FANTOM5 CAGE data for reference.

S2 Table. Counts of female and male FANTOM 5 CAGE samples associated with cell ontology terms.

S3 Table. Differential transcription analyses of TSSs between sexes using FANTOM5 CAGE data.
Table A lists all TSSs on chrX in a decreasing order of significance of assessed differential transcription between sexes. Table B lists the TSSs on autosomes with Bonferroni-corrected p-values under 0.05 in a decreasing order of significance in differential transcription between sexes. The autosomal TSS list is significantly over-represented with repetitive elements, for which alignment biases could be contributing to the difference (see S1 File).

S1 File. Supplementary text file.

S1 Fig. Illustrated dataset properties on chrX in both sexes.
Figure (**A**) and **(B)** illustrate the theoretical transcription and DNA methylation levels at TSSs of two subject genes (S), a bi-allelically transcribed escapee (Bi-E) and *XIST* comparing female and male cells. The bottom track shows the sex comparison with data above or below the horizontal line indicating higher level in male or female, respectively. In general, 'Bi-E' TSSs exhibit higher transcription level in female than male cells, while having similar DNAm levels between sexes. 'S' TSSs share similar transcription levels

between sexes, but higher DNAm level in female cells. The *XIST* TSS is mainly expressed in female cells, and has higher DNAm level in male cells. Figure (**C**) illustrates the ChIP-seq and input reads at TSSs of the same genes in female and male cells. In general, the overall ChIP-seq read depth of a positive regulator in female cells is higher at 'Bi-E' than 'S' TSSs due to its bi-allelic activity. With only one chrX in male cells, such difference is not expected. Figure (**D**) illustrates the compilation of allelic read counts at where heterozygous sites are present. Xa-biased binding at 'S' (H1), relatively balanced binding at 'Bi-E' (H2), and Xi-biased binding at *XIST* (H3) are expected for a positive regulator. Heterozygous sites (such as H4) not within the binding peak of the positive regulatory are not informative. Regions without a heterozygous site are missing data.

S2 Fig. Experimental and GRO-seq data support for escTSSs.
Figures (**A**) and (B) show the qPCR and public GRO-seq data for the escTSS (chrX:41194262-41194297,-) that is antisense to the *DDX3X* gene. Figure (**A**) shows the ratio of *DDX3X-AS* to *ACTIN* as detected by qPCR. The somatic cell hybrid cell lines AHA-11aB1 and t60-12 contain the mouse genome along with a human active X chromosome. T11-4Aaz5, t75-2maz34-4a and t86-B1maz1b-3a contain a mouse genome along with a human inactive X chromosome and between one and ten other human chromosomes but not including an active X chromosome. The mouse parental cell line, tsA1S9-az31b was used as a control to ensure that the *DDX3X-AS* primers were human specific, while the *ACTIN* primers were not human specific. The Y-axis is two to the power of the CT difference between *DDX3X-AS* and *ACTIN* for each cell line. Figure (**B**) shows the GRO-seq data from three female cell lines (IMR90, K562 and GM12878). Figure (**C**) lists the information on GRO-seq and experimental support for each of the five novel escTSSs.

S3 Fig. DNAm comparison between sexes in samples from three other cancers.
The file provides the same plot as Fig 2 for the three additional cancer types, in the order of Colon adenocarcinoma (COAD), Head and Neck squamous cell carcinoma (HNSC), Lung adenocarcinoma (LUAD). (**A**) DNA methylation status for positions (i.e. probes from the Illumina 450k array) near TSSs in both sexes from cancer samples, where the β values (Y-axis) range from 0 (unmethylated) to 1 (fully methylated). The three TSSs are most proximal to the following genes (from top to bottom): *XIST*, an escapee (*ZFX*) and a subject gene (*HMGB3*). Each square represents a sample for the cancer dataset. Red or blue color represents a female or male sample, respectively. Each violin plot in gray lines shows the distributions of beta values for each sex at each probe. Plots (**B**) and (**C**) show MA plots for chrX probes and autosomal probes on chr7 between sexes, respectively. Each dot represents a probe from the array. M (difference) on y-axis is the logged differential methylation value between sexes, and A (magnitude) on x-axis is the logged average methylation value (as indicated in Methods). The fitted robust regression line is represented in gray, with the corresponding function and correlation reported. Green and red colors in plot (**B**) represent probes nearest to escapees and subject genes previously reported in Cotton *et al*. 2015. Gold and gray colors represent probes nearest to *XIST* and genes not in either three categories. (**D**) Violin plots showing the distributions of DNA methylation similarity scores between sexes for probes within 50bps of escTSSs and non-differentially transcribed (nonDT) TSSs on chrX. The similarity score of DNA

methylation on y-axis is the residual of M as a function of A on chrX. Only TSSs with at least one probe within 50bps were plotted, and for those TSSs within 50bps of multiple probes, the average similarity scores of probes were obtained. The p-value from the Wilcoxon test is reported.

S4 Table. Motif over-representation around escTSSs reported by the CAGEdoPOSSUM tool.
Table A and B list all 478 motifs for vertebrates from JASPAR 2016 with the corresponding Fisher scores per motif compared to two background sets, respectively: escTSSs_bg and nonDT.

S5 Table. TF ChIP-seq peak over-representation testing around escTSSs.
The table lists the significance of over-representation for each ChIP-seq data compared to both X_bg and Auto_bg backgrounds. The table is ordered according to the significance compared to X_bg.

S4 Fig. Motifs and ENCODE ChIP-seq peaks of YY1, Myc and CTCF around top escTSSs and superloop-associated lncRNAs.
Figures show 500bps up- and down-stream regions for seven of the top escapees and heterozygous sites with significant Xi-biased YY1 binding in the four lncRNAs. Only motifs with a minimum score of 85% are shown.

S6 Table. TF ChIP-seq peak over-representation testing around TSSs of escapees from X;autosome translocation studies.
The table lists the significance of over-representation for each ChIP-seq data comparing TSSs of escapees to TSSs of subjects reported in the literature. The table is ordered according to the significance.

S7 Table. Allelic reads in YY1 ChIP-seq on Xi and Xa at heterozygous sites within YY1 peaks in the GM12878 cell line.
The table listed 67 heterozygous sites in GM12878 that were within uniformly processed YY1 ChIP-seq peaks from UCSC. Allelic read counts reported were sum of reads from the duplicated data generated by HudsonAlpha Institute for Biotechnology.

S8 Table. Allelic reads at heterozygous sites within merged TF peaks in all ChIP-seq data available in the GM12878 cell line.

S5 Fig. Combined allelic imbalance of all 1321 heterozygous sites within merged TF peaks on chrX in GM12878.
Scatter plot showing the log2 ratios of total number of reads on Xa relative to Xi at 1321 heterozygous sites within merged TF peaks from a total of 101 ChIP-seq and DNase I data sets generated by the ENCODE consortium from the GM12878 cell line. Where replicates were provided, reads at Xi and Xa were summed separately. Datasets, represented as squares, are arranged in increasing order of log2 ratio. The dashed line displays the baseline where there is no overall allelic imbalance. The names of outlying

data sets, those exceeding ±2 standard deviations from the mean, are labeled. Each square/data is colored in quartiles of the total read counts at all 1321 heterozygous sites.

S9 Table. Significance of allelic imbalance from Fisher's exact test and FDR correction.

The table lists pairs of heterozygous sites and datasets with significant allelic imbalance. Positive or negative log2Odds values indicate Xa- or Xi- biased occupancy of the data at the corresponding heterozygous site, respectively.

S1 File-Additional text

Cross-reactivity bias of differentially transcribed TSSs and differentially methylated probes on the autosomes

In addition to chrX, sex-specific expression has been previously identified on autosomes in human pancreatic islets, primary T cells, peripheral blood, and brain [1-4]. Applying our differential transcription analysis to autosomes, we found 48 TSSs that were significantly differentially transcribed between sexes on the autosomes using CAGE data (Bonferroni-corrected p-values ≤ 0.05; Supplementary Table S3B). However, these autosomal TSSs were significantly enriched with repetitive elements (21 out of 61 as opposed to 10 out of 103 TSSs on chrX without the repetitive TSS filter; one-sided Fisher's Exact Test p= 0.0001).

The GTEx study also reported differentially expressed autosomal genes between sexes [5], and the only common gene we found was *FRG1B*. An overlap between the nearest genes to both differentially transcribed TSSs and differential methylated probes across four cancer types (see methods) revealed two autosomal genes, *FRG1B* and *PSMA6*. However, probes for the two genes were confounded with cross-reactivity reported in Price *et al.* [6]. The *PSMA6* probes aligned to the sex chromosomes, whereas *FRG1B* probes can align to multiple places on autosomes. The significant overlap with repetitive elements and cross-reactivity collectively revealed potential bias in conducting sex analysis on the autosomes.

Cross-reaction and multiple aligning hits to the genome of probes in Illumina 450k DNA methylation arrays reported in the literature [6-8] can induce bias to sex analyses, especially when autosomal TSSs or probes align to sex chromosomes. Due to cross-reactivity of probes and the significant enrichment in overlapping repetitive elements, we found our CAGE and DNA methylation analyses on autosomes to be strongly biased. Despite previous success in identifying sex-specific expression on autosomes with relevant functions, the differentially transcribed autosomes TSSs we identified using CAGE datasets significantly overlapped with repetitive elements or had cross-reactivity to multiple places in the genome when compared to differentially methylated probes. We note that the successful studies focused on specific cell types, whereas our model was geared to identify differentially expressed autosomal gene across cell types. This is consistent with the previous report of substantial tissue specificity of sex-biased autosomal genes [3]. Interaction terms between sex and cell category covariates can be introduced to identify cell type-specific sex differences given sufficient number of samples.

Overall Xa-biased occupancy in the female GM12878 cell line

We computed the log2 ratio of the total number of reads on Xa to Xi at 1321 heterozygous sites within peaks in all replicate-merged datasets and found an overall higher number of reads on Xa compared to Xi (Supplementary Fig. S5). The inactive histone mark, H3K27me3, was strongly biased toward Xi binding (with log2 ratio less than overall mean minus 2 standard deviations), whereas PML, RUNX3, NFATC1 and NFIC were strongly biased toward Xa binding. We also observed a lower number of

reads overall from negative regulators, which could be in part due to the lack of accessibility in inactive regions or experimental bias.

Reference

1 Qu, K. *et al.* Individuality and variation of personal regulomes in primary human T cells. *Cell Syst* **1**, 51-61, doi:10.1016/j.cels.2015.06.003 (2015).

2 Hall, E. *et al.* Sex differences in the genome-wide DNA methylation pattern and impact on gene expression, microRNA levels and insulin secretion in human pancreatic islets. *Genome Biol* **15**, 522, doi:10.1186/s13059-014-0522-z (2014).

3 Jansen, R. *et al.* Sex differences in the human peripheral blood transcriptome. *BMC Genomics* **15**, 33, doi:10.1186/1471-2164-15-33 (2014).

4 Trabzuni, D. *et al.* Widespread sex differences in gene expression and splicing in the adult human brain. *Nat Commun* **4**, 2771, doi:10.1038/ncomms3771 (2013).

5 The Genotype-Tissue Expression (GTEx) pilot analysis: Multitissue gene regulation in humans. *Science* **348**, 648-660, doi:10.1126/science.1262110 (2015).

6 Price, M. E. *et al.* Additional annotation enhances potential for biologically-relevant analysis of the Illumina Infinium HumanMethylation450 BeadChip array. *Epigenetics Chromatin* **6**, 4, doi:10.1186/1756-8935-6-4 (2013).

7 Naeem, H. *et al.* Reducing the risk of false discovery enabling identification of biologically significant genome-wide methylation status using the HumanMethylation450 array. *BMC Genomics* **15**, 51, doi:10.1186/1471-2164-15-51 (2014).

8 Chen, Y. A. *et al.* Discovery of cross-reactive probes and polymorphic CpGs in the Illumina Infinium HumanMethylation450 microarray. *Epigenetics* **8**, 203-209, doi:10.4161/epi.23470 (2013).

Supplementary Fig. S1

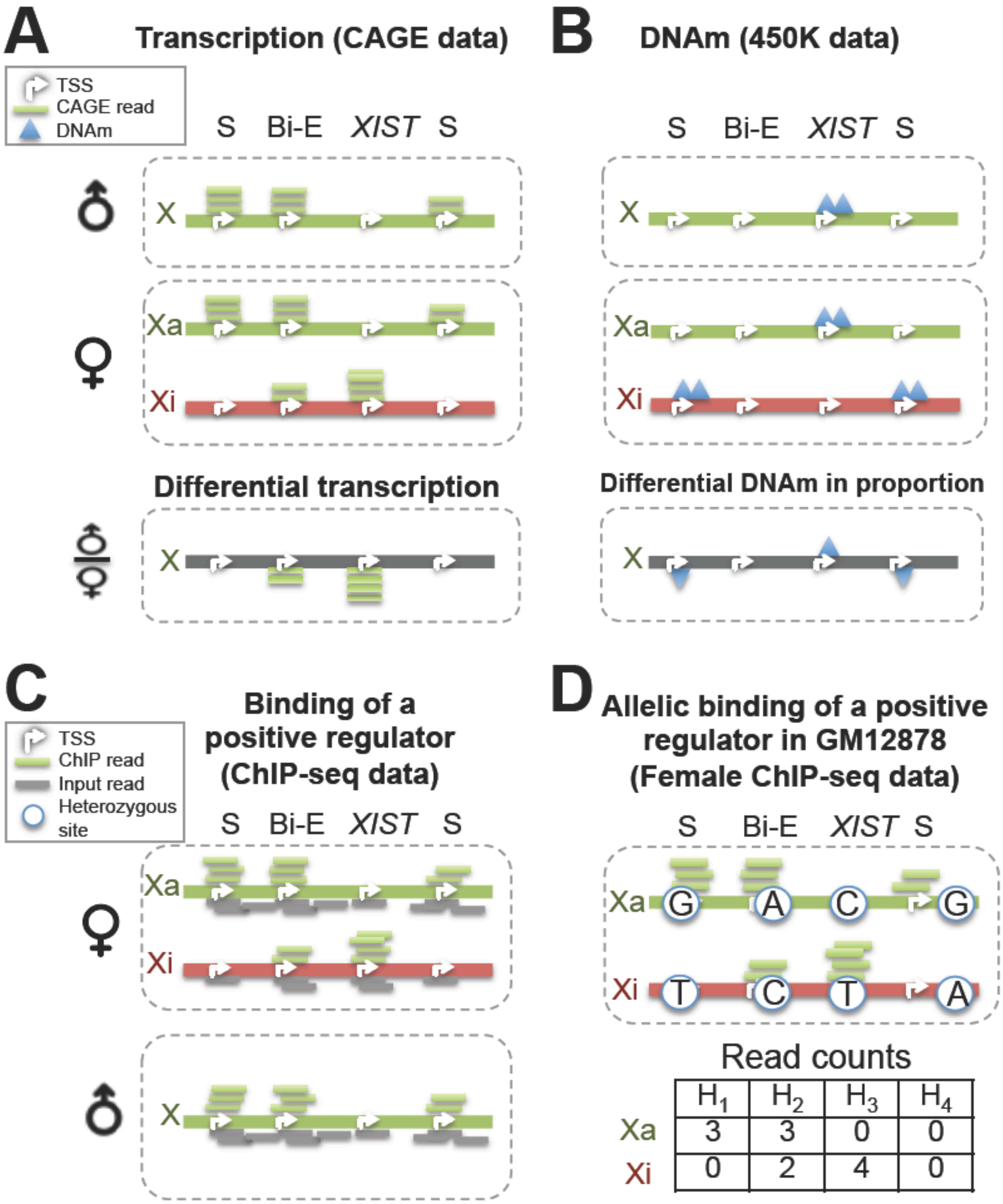

	H_1	H_2	H_3	H_4
Xa	3	3	0	0
Xi	0	2	4	0

Supplementary Fig. S2

A

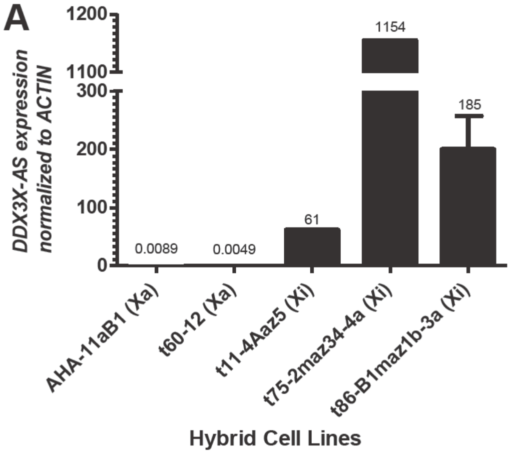

Primers:

DDX3X T7 -tagged strand specific primer
TAATACGACTCACTATAGGGAGAACA
ATAGCAACCAACTCCCC

DDX3X amplification primer
CCAGAAAGTAATCTTAAGCCATCTAG

ACTIN T7 -tagged strand specific primer
TAATACGACTCACTATAGGGAGAACT
GGGACGACATGGAGAAAA

ACTIN amplification primer
GCCACACGCAGCTCATTGT

T7 primer
TAATACGACTCACTATAGGGAGA

B

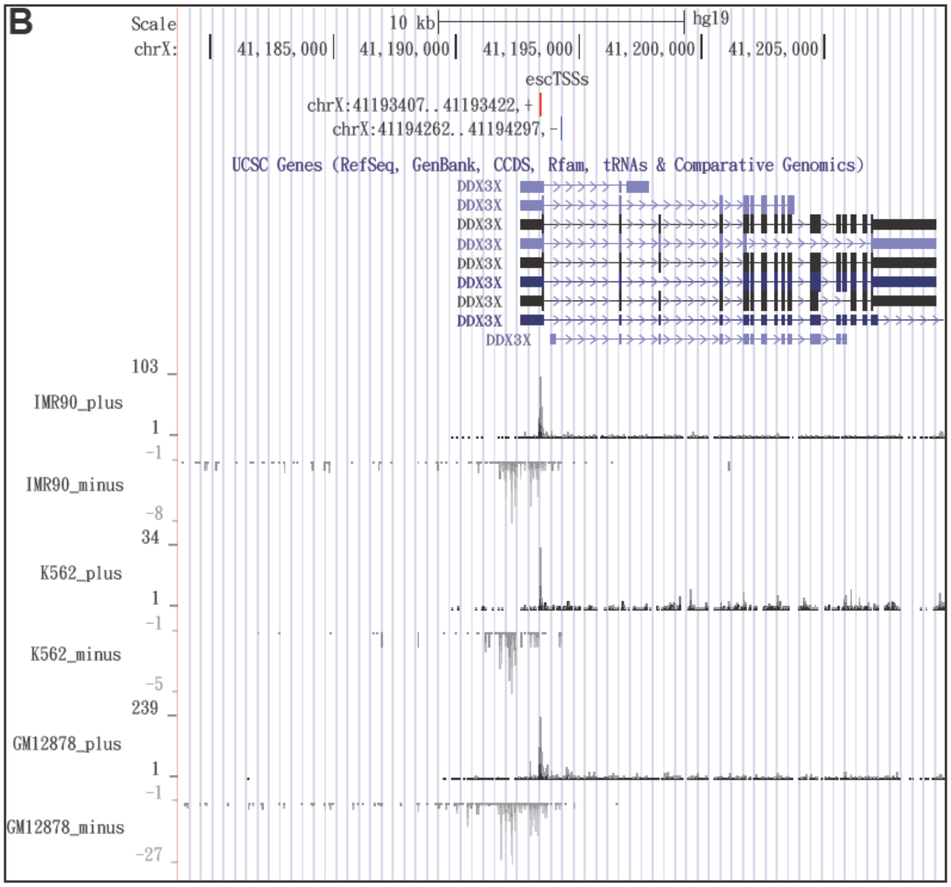

C

CAGE peak(s) coordinates	Nearest Gene	Antisense to Gene	GRO-seq	Strand-specific RT-qPCR validation
chrX:41194262-41194297,-	*RN7SL15P*	*DDX3X*	Yes	Yes (Xi-specific expression)
chrX:73040494-73040497,+	*TSIX*	*XIST*	No	No amplification
chrX:7065835-7065770,+<br>chrX:7065704-7065908,+	*MIR4767 / STS*	*HDHD1*	Yes	--<br>(High GC region*)
chrX:3241475-3241523,+	*ASS1P4*	*MXRA5*	No	No amplification

Supplementary Fig. S3

COAD

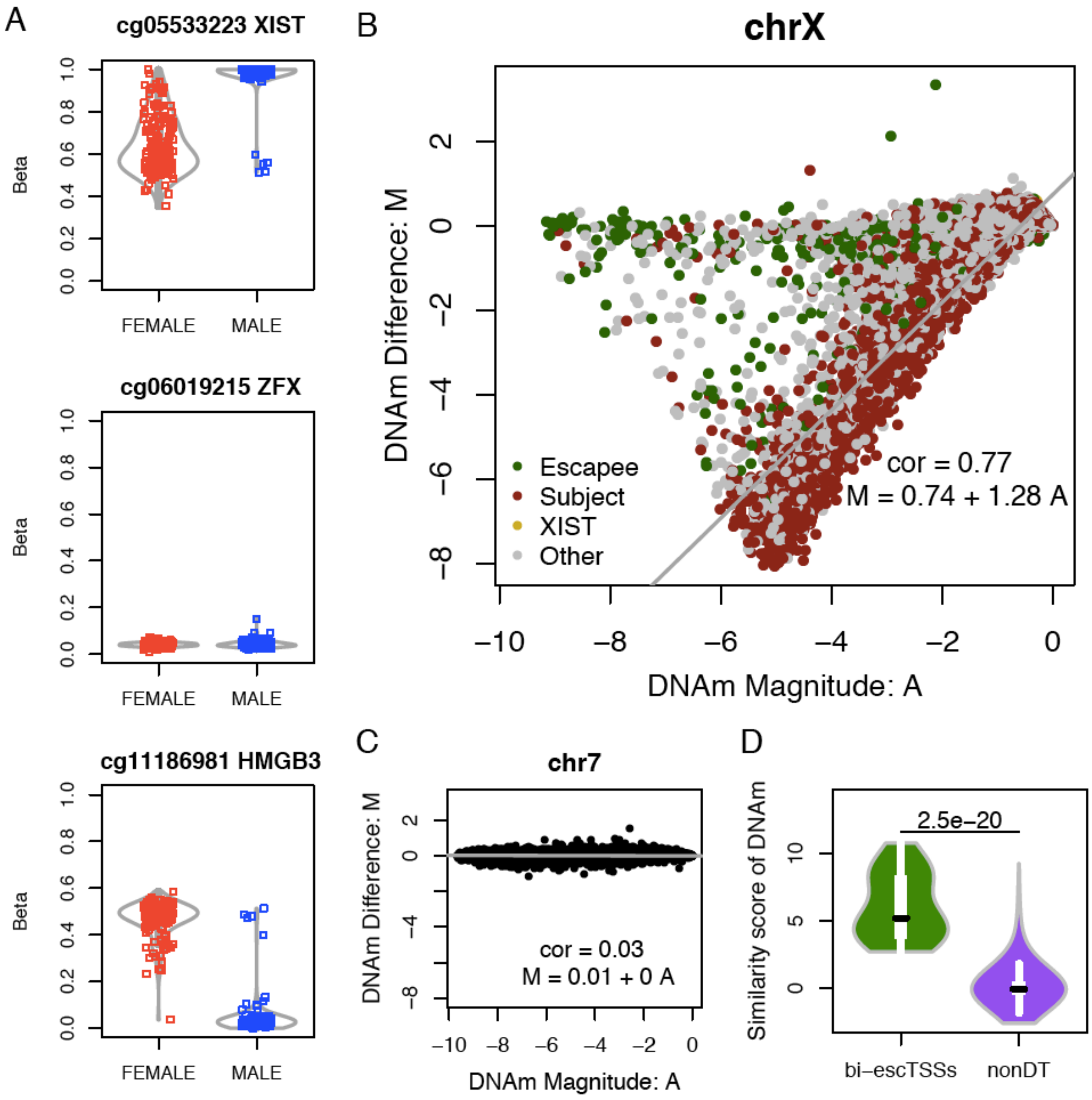

HNSC

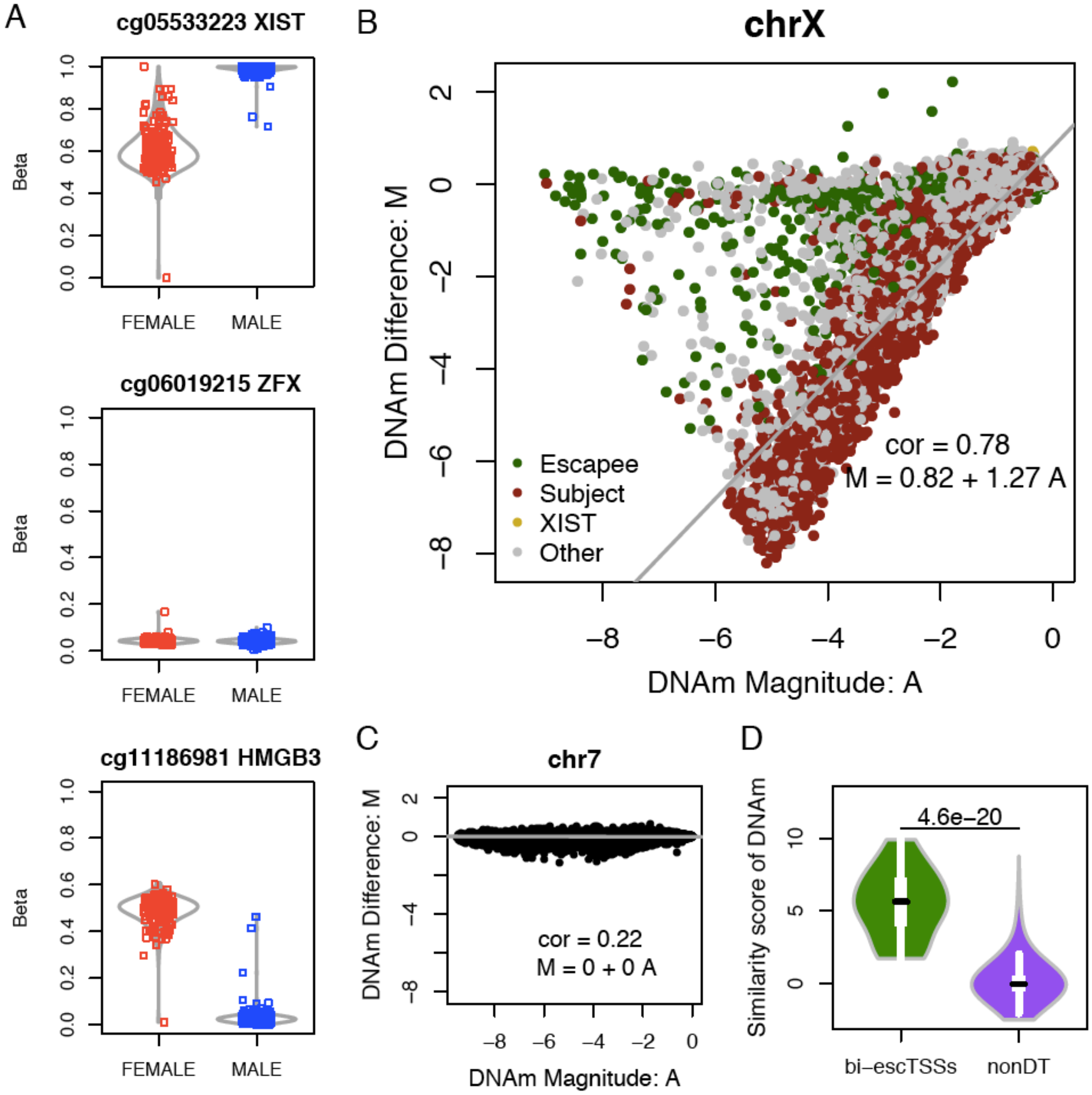
A
cg05533223 XIST
Beta
FEMALE
MALE
cg06019215 ZFX
Beta
FEMALE
MALE
cg11186981 HMGB3
Beta
FEMALE
MALE
B
chrX
DNAm Difference: M
DNAm Magnitude: A
Escapee
Subject
XIST
Other
cor = 0.78
M = 0.82 + 1.27 A
C
chr7
DNAm Difference: M
DNAm Magnitude: A
cor = 0.22
M = 0 + 0 A
D
Similarity score of DNAm
4.6e−20
bi−escTSSs
nonDT

LUAD

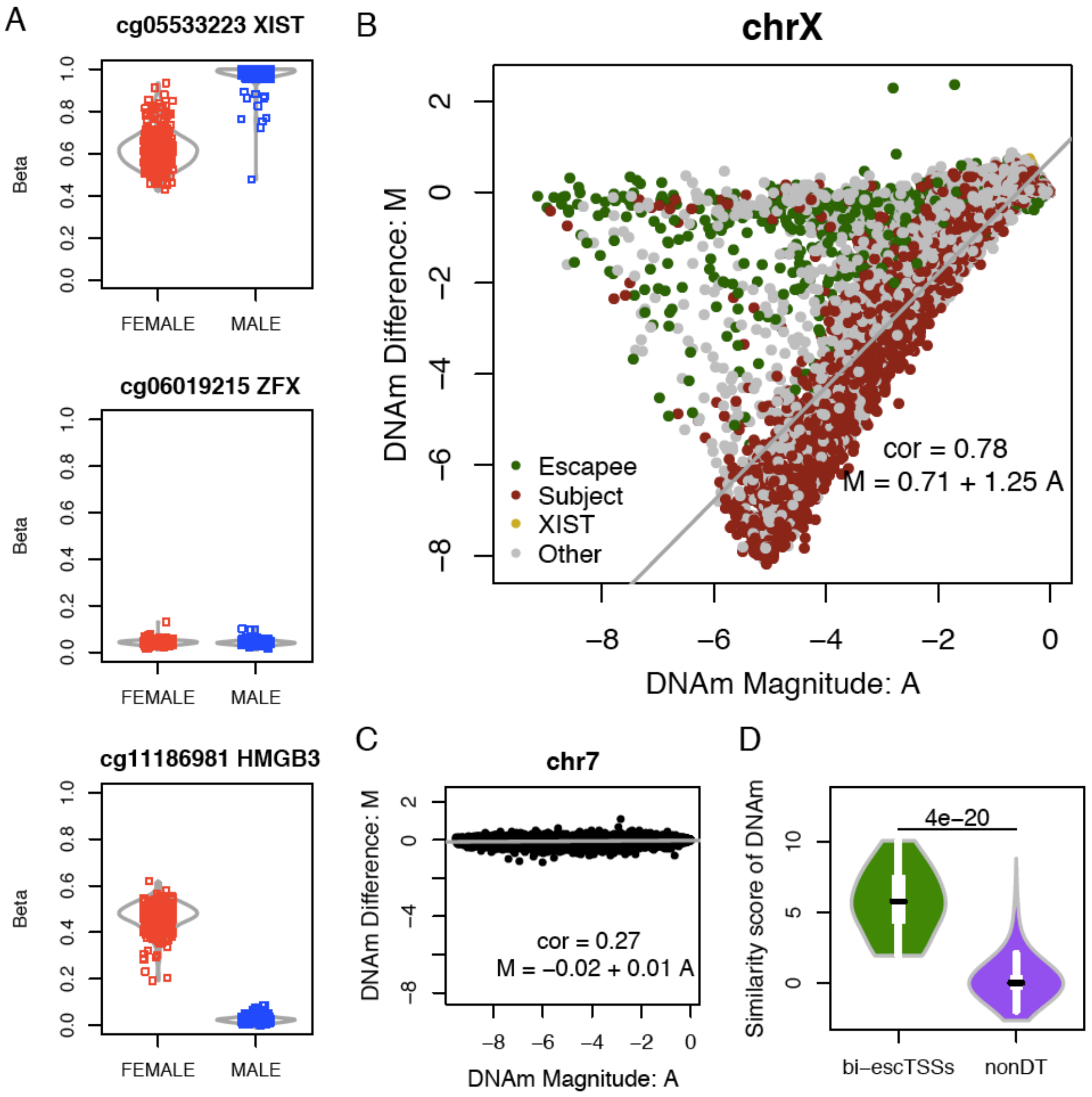
A
cg05533223 XIST
Beta
FEMALE
MALE
cg06019215 ZFX
cg11186981 HMGB3
B
chrX
DNAm Difference: M
DNAm Magnitude: A
Escapee
Subject
XIST
Other
cor = 0.78
M = 0.71 + 1.25 A
C
chr7
cor = 0.27
M = −0.02 + 0.01 A
D
Similarity score of DNAm
4e−20
bi−escTSSs
nonDT

Supplementary Fig. S4

escTSSs

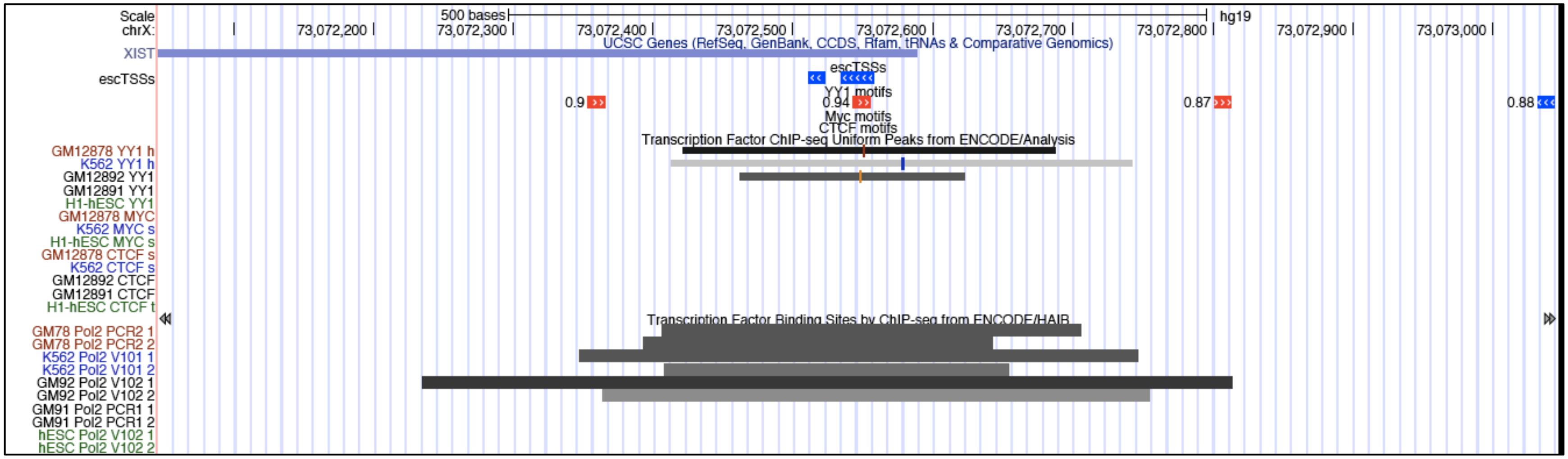

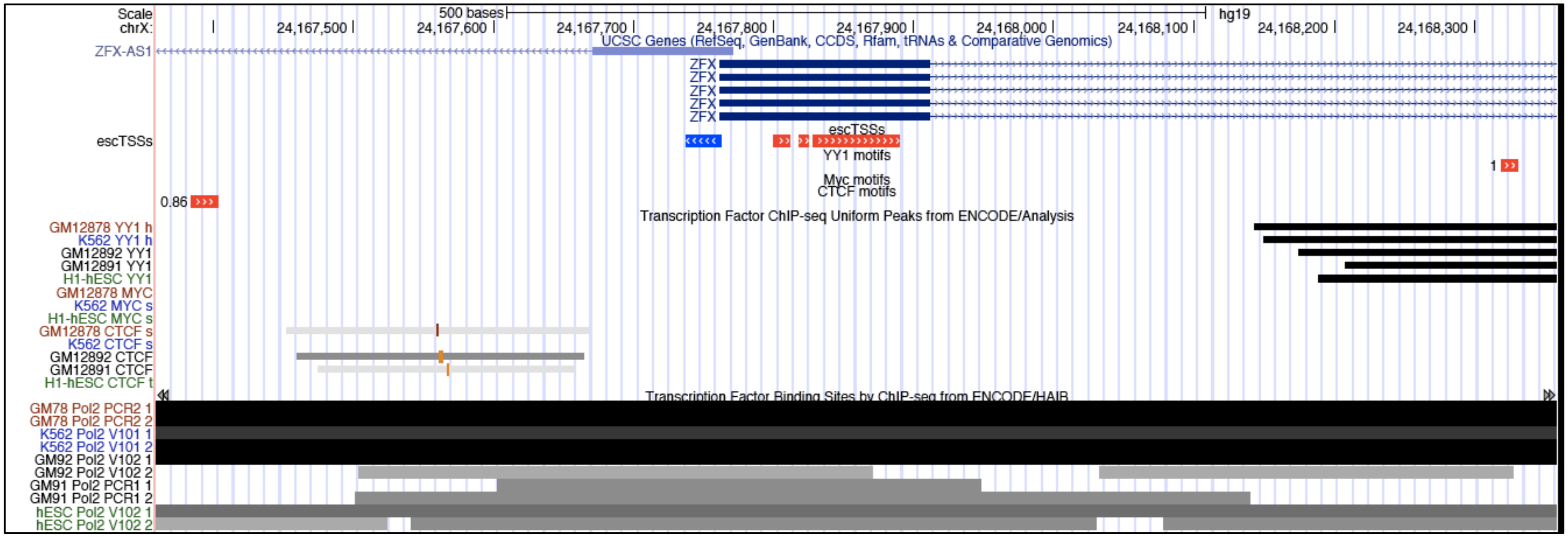

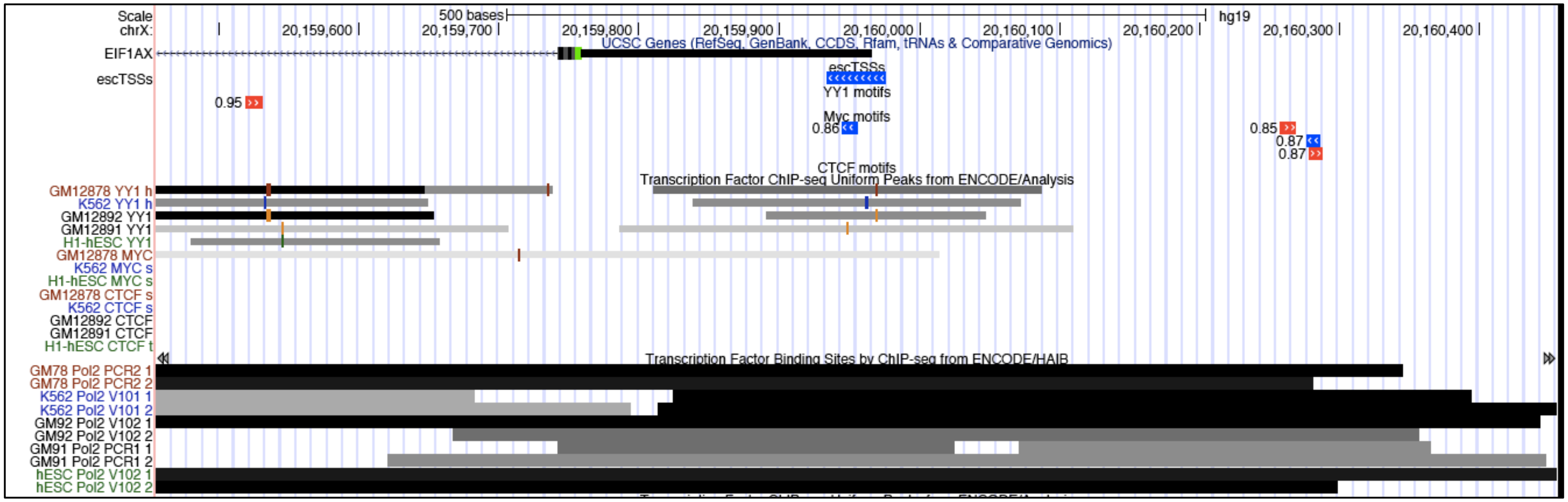
Scale
chrX:
500 bases
hg19
20,159,600
20,159,700
20,159,800
20,159,900
20,160,000
20,160,100
20,160,200
20,160,300
20,160,400
EIF1AX
UCSC Genes (RefSeq, GenBank, CCDS, Rfam, tRNAs & Comparative Genomics)
escTSSs
escTSSs
YY1 motifs
0.95
Myc motifs
0.86
0.85
0.87
0.87
CTCF motifs
Transcription Factor ChIP-seq Uniform Peaks from ENCODE/Analysis
GM12878 YY1 h
K562 YY1 h
GM12892 YY1
GM12891 YY1
H1-hESC YY1
GM12878 MYC
K562 MYC s
H1-hESC MYC s
GM12878 CTCF s
K562 CTCF s
GM12892 CTCF
GM12891 CTCF
H1-hESC CTCF t
Transcription Factor Binding Sites by ChIP-seq from ENCODE/HAIB
GM78 Pol2 PCR2 1
GM78 Pol2 PCR2 2
K562 Pol2 V101 1
K562 Pol2 V101 2
GM92 Pol2 V102 1
GM92 Pol2 V102 2
GM91 Pol2 PCR1 1
GM91 Pol2 PCR1 2
hESC Pol2 V102 1
hESC Pol2 V102 2

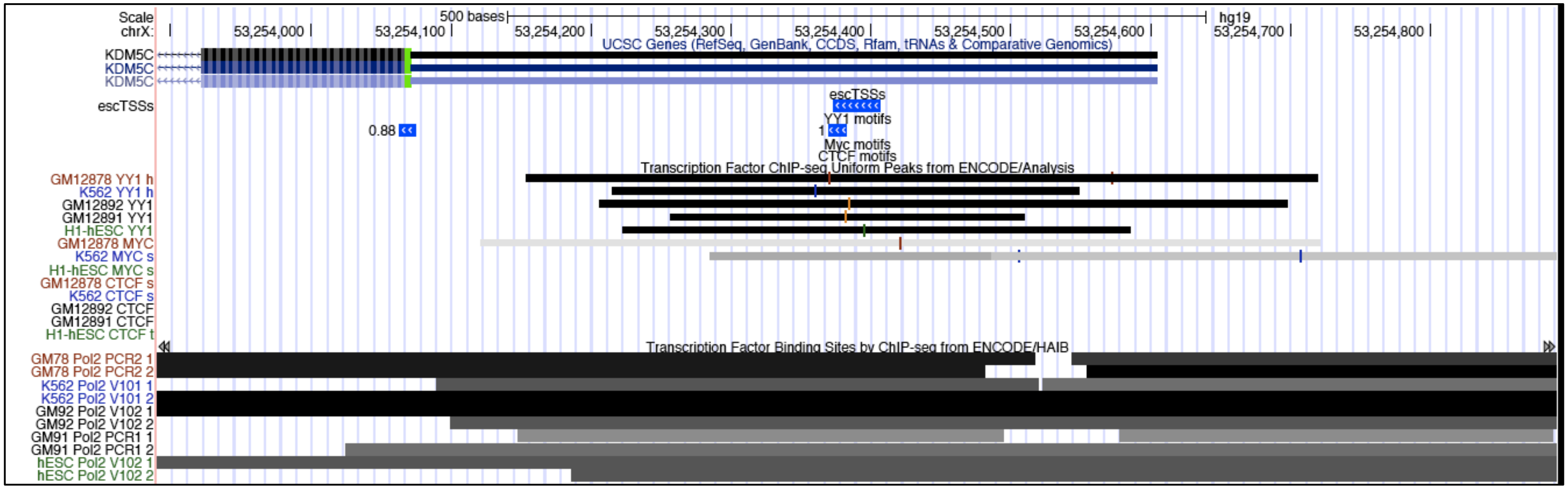
Scale
chrX:
500 bases
hg19
53,254,000
53,254,100
53,254,200
53,254,300
53,254,400
53,254,500
53,254,600
53,254,700
53,254,800
KDM5C
KDM5C
KDM5C
UCSC Genes (RefSeq, GenBank, CCDS, Rfam, tRNAs & Comparative Genomics)
escTSSs
escTSSs
YY1 motifs
0.88
1
Myc motifs
CTCF motifs
Transcription Factor ChIP-seq Uniform Peaks from ENCODE/Analysis
GM12878 YY1 h
K562 YY1 h
GM12892 YY1
GM12891 YY1
H1-hESC YY1
GM12878 MYC
K562 MYC s
H1-hESC MYC s
GM12878 CTCF s
K562 CTCF s
GM12892 CTCF
GM12891 CTCF
H1-hESC CTCF t
Transcription Factor Binding Sites by ChIP-seq from ENCODE/HAIB
GM78 Pol2 PCR2 1
GM78 Pol2 PCR2 2
K562 Pol2 V101 1
K562 Pol2 V101 2
GM92 Pol2 V102 1
GM92 Pol2 V102 2
GM91 Pol2 PCR1 1
GM91 Pol2 PCR1 2
hESC Pol2 V102 1
hESC Pol2 V102 2

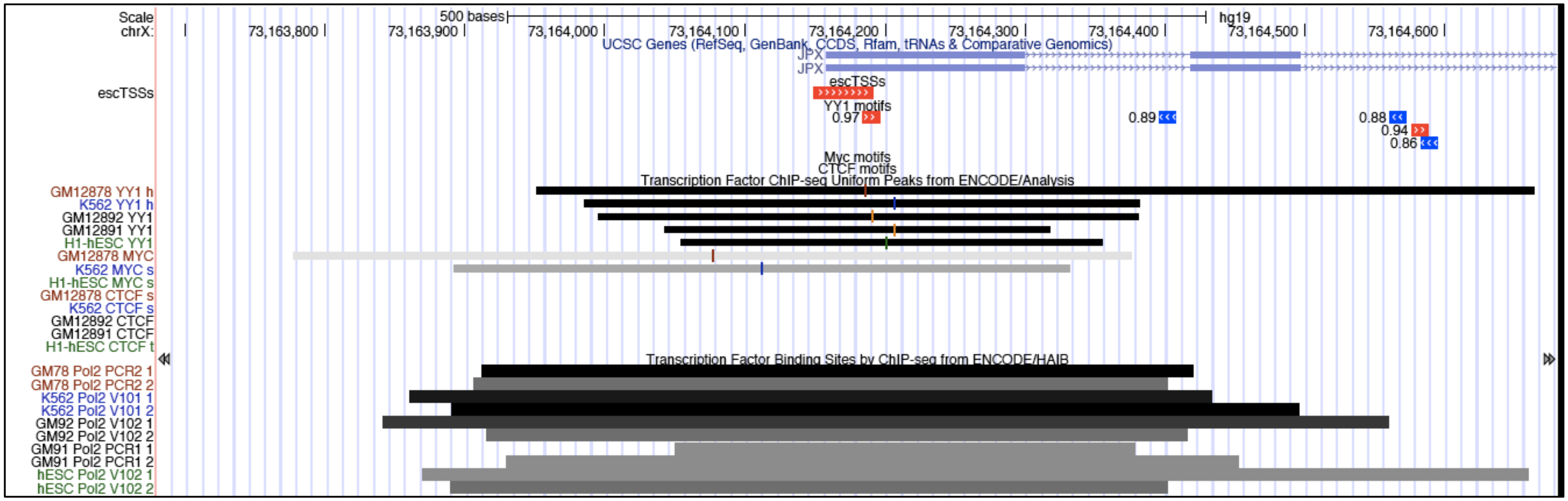
Scale
chrX:
500 bases
hg19
73,163,800
73,163,900
73,164,000
73,164,100
73,164,200
73,164,300
73,164,400
73,164,500
73,164,600
UCSC Genes (RefSeq, GenBank, CCDS, Rfam, tRNAs & Comparative Genomics)
JPX
JPX
escTSSs
escTSSs
YY1 motifs
0.97
0.89
0.88
0.94
0.86
Myc motifs
CTCF motifs
Transcription Factor ChIP-seq Uniform Peaks from ENCODE/Analysis
GM12878 YY1 h
K562 YY1 h
GM12892 YY1
GM12891 YY1
H1-hESC YY1
GM12878 MYC
K562 MYC s
H1-hESC MYC s
GM12878 CTCF s
K562 CTCF s
GM12892 CTCF
GM12891 CTCF
H1-hESC CTCF t
Transcription Factor Binding Sites by ChIP-seq from ENCODE/HAIB
GM78 Pol2 PCR2 1
GM78 Pol2 PCR2 2
K562 Pol2 V101 1
K562 Pol2 V101 2
GM92 Pol2 V102 1
GM92 Pol2 V102 2
GM91 Pol2 PCR1 1
GM91 Pol2 PCR1 2
hESC Pol2 V102 1
hESC Pol2 V102 2

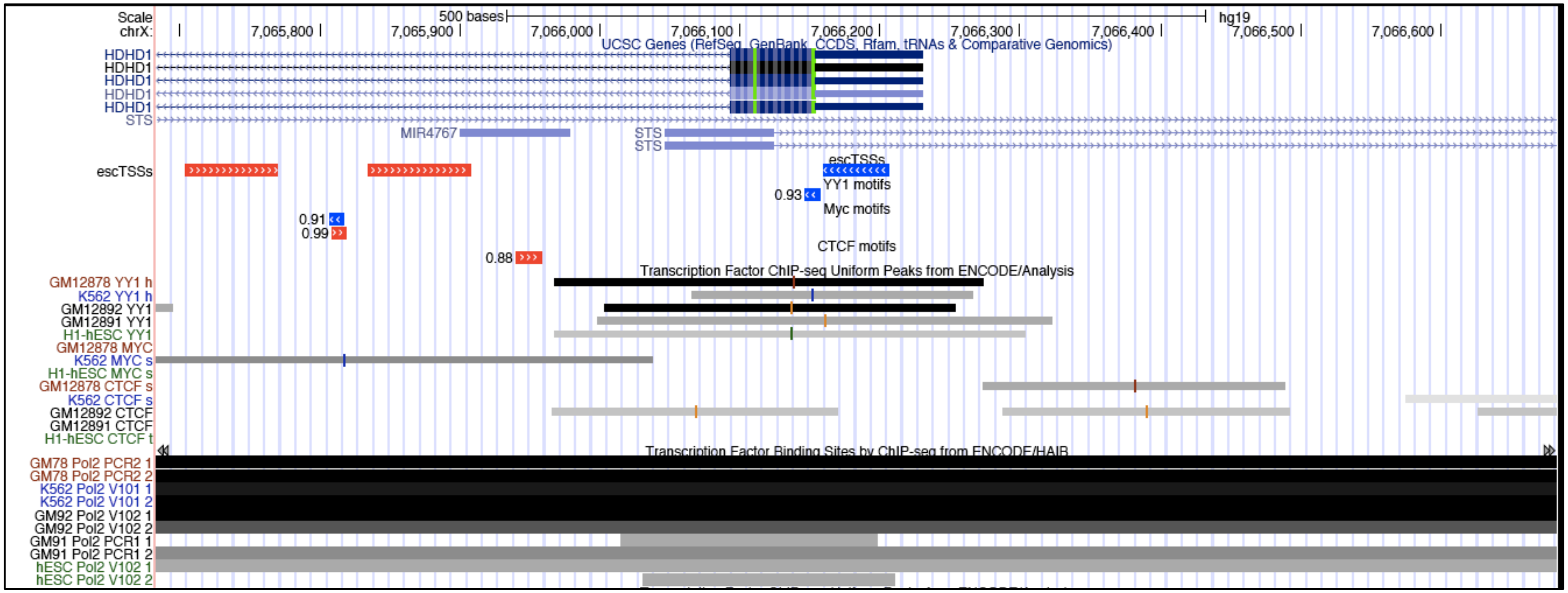
Scale
chrX:
500 bases
hg19
7,065,800
7,065,900
7,066,000
7,066,100
7,066,200
7,066,300
7,066,400
7,066,500
7,066,600
UCSC Genes (RefSeq, GenBank, CCDS, Rfam, tRNAs & Comparative Genomics)
HDHD1
HDHD1
HDHD1
HDHD1
HDHD1
STS
MIR4767
STS
STS
escTSSs
escTSSs
YY1 motifs
0.93
Myc motifs
0.91
0.99
0.88
CTCF motifs
Transcription Factor ChIP-seq Uniform Peaks from ENCODE/Analysis
GM12878 YY1 h
K562 YY1 h
GM12892 YY1
GM12891 YY1
H1-hESC YY1
GM12878 MYC
K562 MYC s
H1-hESC MYC s
GM12878 CTCF s
K562 CTCF s
GM12892 CTCF
GM12891 CTCF
H1-hESC CTCF t
Transcription Factor Binding Sites by ChIP-seq from ENCODE/HAIB
GM78 Pol2 PCR2 1
GM78 Pol2 PCR2 2
K562 Pol2 V101 1
K562 Pol2 V101 2
GM92 Pol2 V102 1
GM92 Pol2 V102 2
GM91 Pol2 PCR1 1
GM91 Pol2 PCR1 2
hESC Pol2 V102 1
hESC Pol2 V102 2

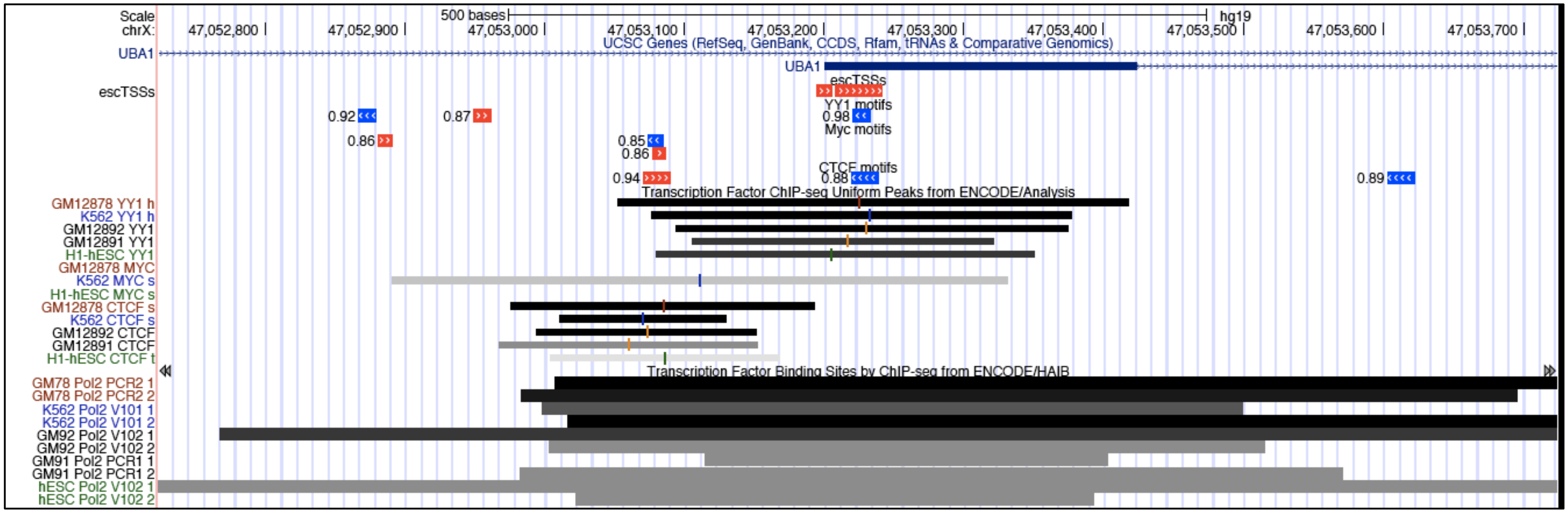

Scale
chrX:
500 bases
hg19
47,052,800
47,052,900
47,053,000
47,053,100
47,053,200
47,053,300
47,053,400
47,053,500
47,053,600
47,053,700
UCSC Genes (RefSeq, GenBank, CCDS, Rfam, tRNAs & Comparative Genomics)
UBA1
UBA1
escTSSs
escTSSs
YY1 motifs
0.92
0.87
0.98
Myc motifs
0.86
0.85
0.86
CTCF motifs
0.94
0.88
0.89
Transcription Factor ChIP-seq Uniform Peaks from ENCODE/Analysis
GM12878 YY1 h
K562 YY1 h
GM12892 YY1
GM12891 YY1
H1-hESC YY1
GM12878 MYC
K562 MYC s
H1-hESC MYC s
GM12878 CTCF s
K562 CTCF s
GM12892 CTCF
GM12891 CTCF
H1-hESC CTCF t
Transcription Factor Binding Sites by ChIP-seq from ENCODE/HAIB
GM78 Pol2 PCR2 1
GM78 Pol2 PCR2 2
K562 Pol2 V101 1
K562 Pol2 V101 2
GM92 Pol2 V102 1
GM92 Pol2 V102 2
GM91 Pol2 PCR1 1
GM91 Pol2 PCR1 2
hESC Pol2 V102 1
hESC Pol2 V102 2

lncRNAs (heterozygous sites)

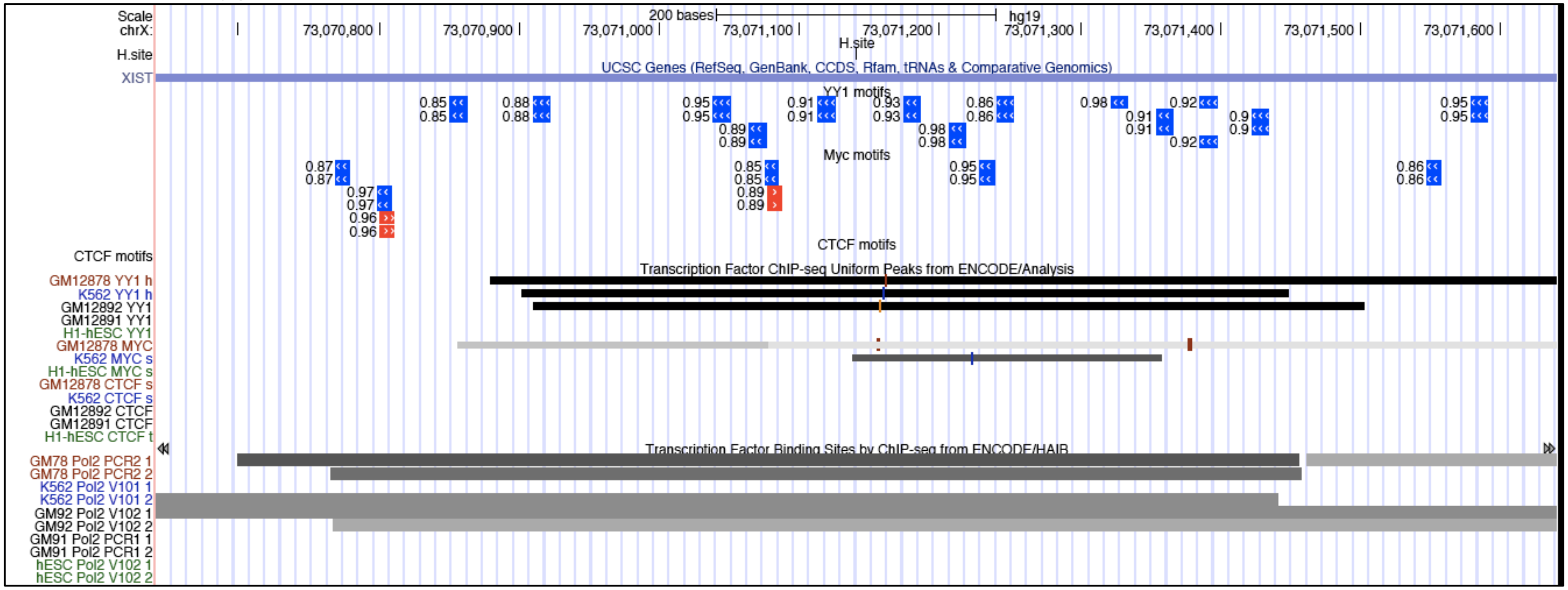

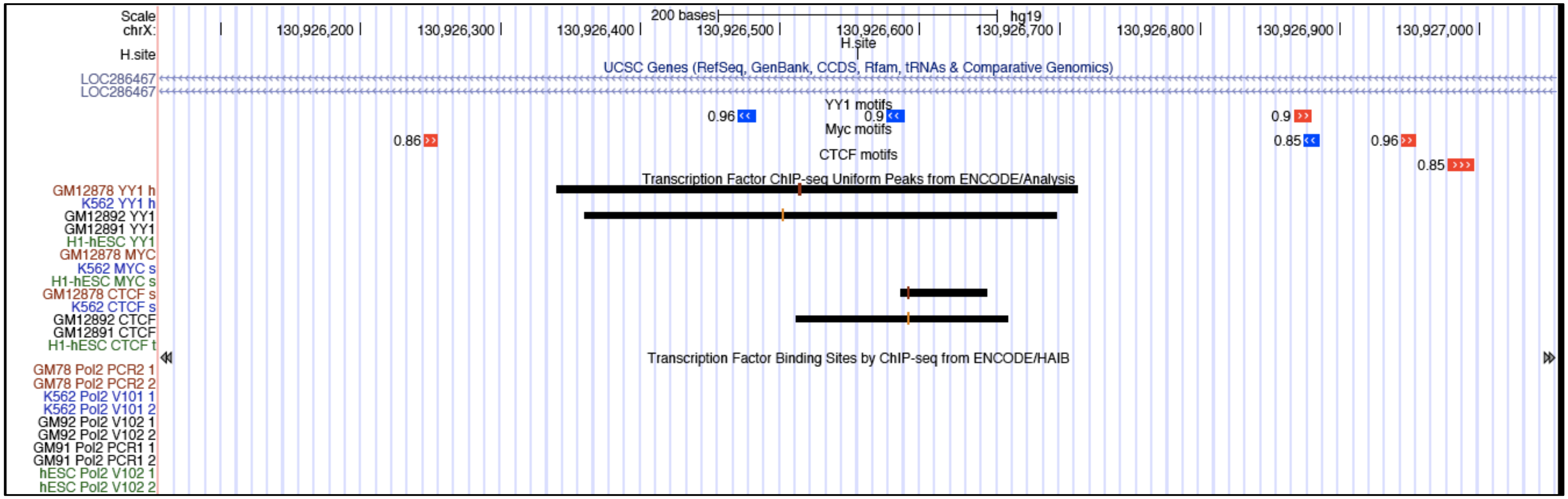

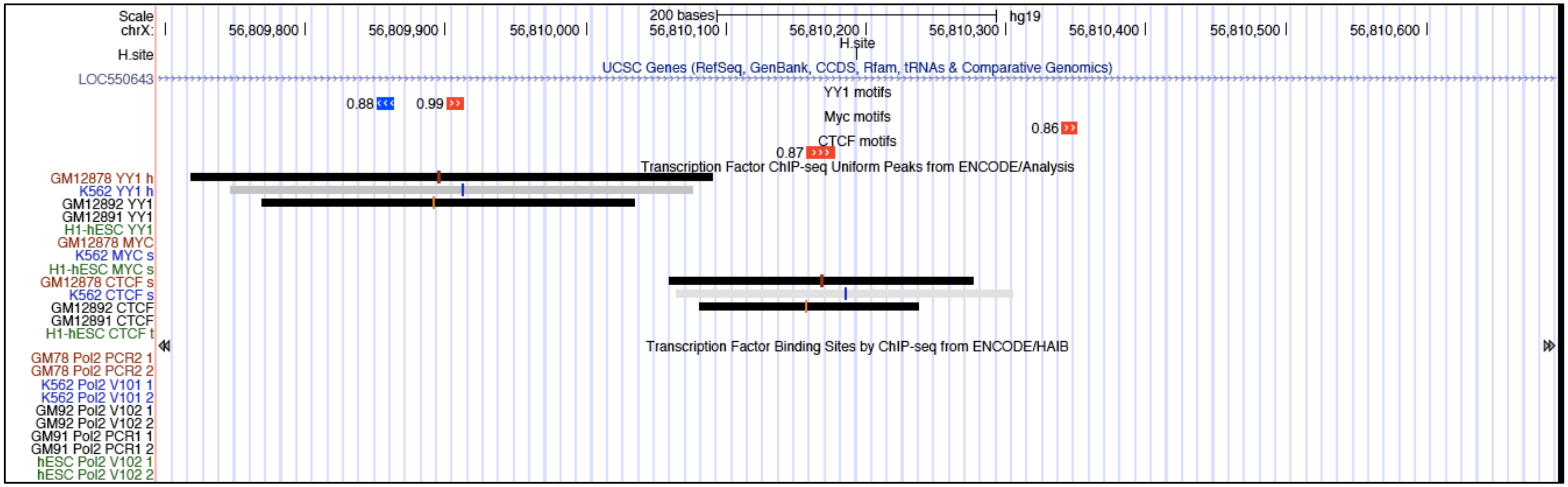

Scale
chrX:
200 bases
hg19
56,809,800
56,809,900
56,810,000
56,810,100
56,810,200
56,810,300
56,810,400
56,810,500
56,810,600
H.site
UCSC Genes (RefSeq, GenBank, CCDS, Rfam, tRNAs & Comparative Genomics)
LOC550643
YY1 motifs
0.88
0.99
Myc motifs
0.86
CTCF motifs
0.87
Transcription Factor ChIP-seq Uniform Peaks from ENCODE/Analysis
GM12878 YY1 h
K562 YY1 h
GM12892 YY1
GM12891 YY1
H1-hESC YY1
GM12878 MYC
K562 MYC s
H1-hESC MYC s
GM12878 CTCF s
K562 CTCF s
GM12892 CTCF
GM12891 CTCF
H1-hESC CTCF t
Transcription Factor Binding Sites by ChIP-seq from ENCODE/HAIB
GM78 Pol2 PCR2 1
GM78 Pol2 PCR2 2
K562 Pol2 V101 1
K562 Pol2 V101 2
GM92 Pol2 V102 1
GM92 Pol2 V102 2
GM91 Pol2 PCR1 1
GM91 Pol2 PCR1 2
hESC Pol2 V102 1
hESC Pol2 V102 2

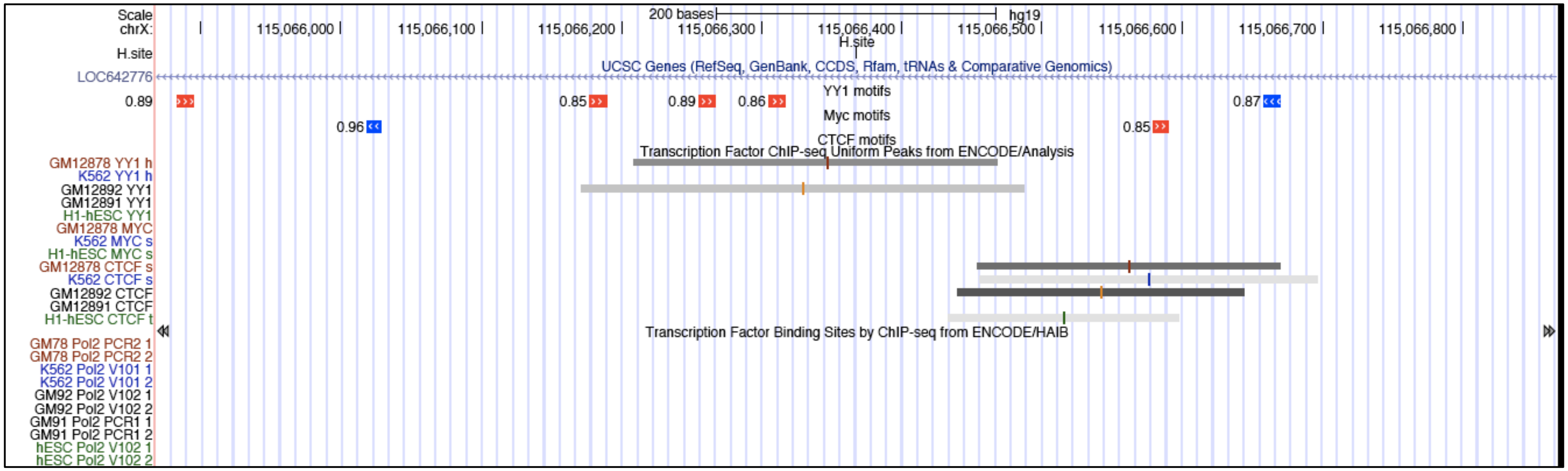

Scale
chrX:
200 bases
hg19
115,066,000
115,066,100
115,066,200
115,066,300
115,066,400
115,066,500
115,066,600
115,066,700
115,066,800
H.site
UCSC Genes (RefSeq, GenBank, CCDS, Rfam, tRNAs & Comparative Genomics)
LOC642776
YY1 motifs
0.89
0.85
0.89
0.86
0.87
Myc motifs
0.96
0.85
CTCF motifs
Transcription Factor ChIP-seq Uniform Peaks from ENCODE/Analysis
GM12878 YY1 h
K562 YY1 h
GM12892 YY1
GM12891 YY1
H1-hESC YY1
GM12878 MYC
K562 MYC s
H1-hESC MYC s
GM12878 CTCF s
K562 CTCF s
GM12892 CTCF
GM12891 CTCF
H1-hESC CTCF t
Transcription Factor Binding Sites by ChIP-seq from ENCODE/HAIB
GM78 Pol2 PCR2 1
GM78 Pol2 PCR2 2
K562 Pol2 V101 1
K562 Pol2 V101 2
GM92 Pol2 V102 1
GM92 Pol2 V102 2
GM91 Pol2 PCR1 1
GM91 Pol2 PCR1 2
hESC Pol2 V102 1
hESC Pol2 V102 2

Supplementary Fig. S5

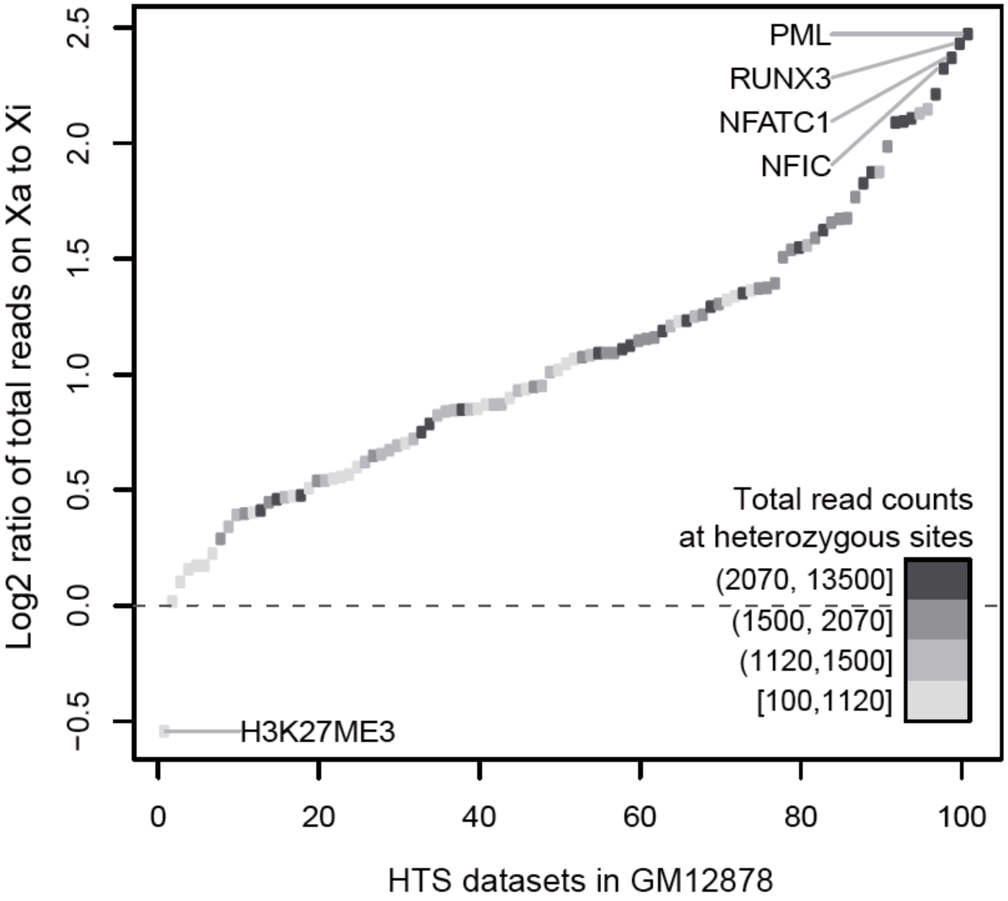

Supplement: Supplementary Information [file srep37324-s1.pdf]
